# FL3 mitigates cardiac ischemia-reperfusion injury by promoting mitochondrial fusion to restore calcium homeostasis

**DOI:** 10.1038/s41420-025-02575-w

**Published:** 2025-07-03

**Authors:** Zikan Zhong, Yutong Hou, Changzuan Zhou, Jiahui Wang, Longzhe Gao, Xiaoyu Wu, Genqing Zhou, Shaowen Liu, Yingjie Xu, Wen Yang

**Affiliations:** 1 Department of Cardiology, Shanghai General Hospital, Shanghai Jiao Tong University School of Medicine, Shanghai, China; 2 Department of Biochemistry and Molecular Cell Biology, Shanghai Key Laboratory for Tumor Microenvironment and Inflammation, Shanghai Jiao Tong University School of Medicine, Shanghai, 200025 PR China; 3 Key Laboratory of Cell Differentiation and Apoptosis of Chinese Ministry of Education, Shanghai Jiao Tong University School of Medicine, Shanghai, 200025 PR China; 4 State Key Laboratory of Oncogenes and Related Genes, Shanghai, PR China

**Keywords:** Target validation, Apoptosis

## Abstract

This study aims to investigate the therapeutic potential of Flavagline3 (FL3) in mitigating myocardial ischemia-reperfusion (IR) injury, with a specific focus on its regulatory effects on mitochondrial fusion, mitochondrial-endoplasmic reticulum (ER) interactions, and calcium homeostasis in cardiomyocytes. Using a well-established myocardial IR injury model in mice and primary cardiomyocytes treated with FL3, the study assessed its impact on mitochondrial dynamics and intracellular signaling processes. The results demonstrated that FL3 effectively reduced myocardial apoptosis, infarct size, and cardiac dysfunction caused by IR injury. Mechanistically, FL3 promoted mitochondrial fusion in a mitofusin1 (MFN1)-dependent manner, preserving mitochondrial function under stress conditions and enhancing cellular resilience. Furthermore, FL3 facilitated mitochondrial-ER crosstalk, which played a critical role in modulating intracellular calcium levels by optimizing the transfer of calcium ions between these two organelles. This balanced regulation of mitochondrial dynamics and calcium homeostasis was associated with improved survival and functionality of cardiomyocytes following IR injury. These findings suggest that FL3 exerts robust cardioprotective effects through its ability to promote mitochondrial fusion, enhance mitochondrial-ER interactions, and maintain calcium homeostasis. As a result, FL3 holds promise as a potential therapeutic agent for reducing myocardial damage and dysfunction associated with IR injury, offering valuable insights into novel approaches for cardioprotection.

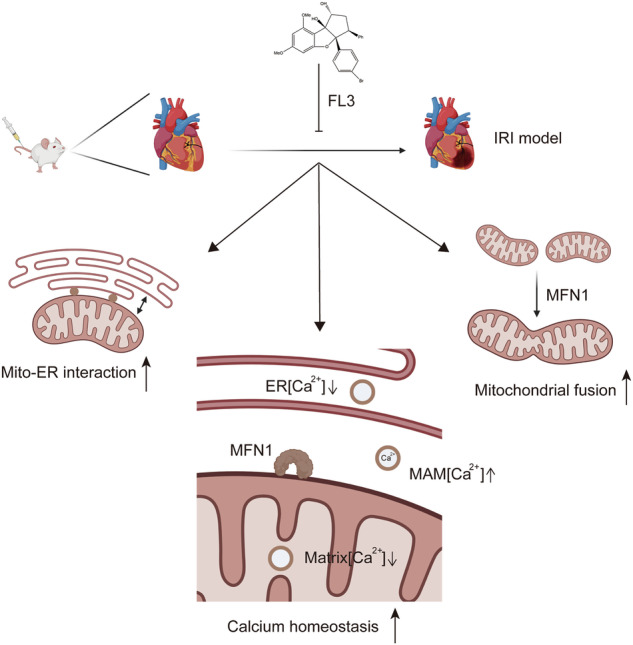

## Introduction

For patients with acute myocardial infarction (AMI), timely reperfusion is essential for salvaging the myocardium and restoring cardiac function. However, reperfusion itself can also lead to myocardial ischemia-reperfusion (IR) injury, which is characterized by local inflammation and an increase in reactive oxygen species (ROS), resulting in additional damage to the myocardium. Despite the benefits of reperfusion therapy, including thrombolysis or primary percutaneous coronary intervention, the complexity of IR injury’s pathogenesis limits current preventive and therapeutic strategies [1].

The primary mechanisms of IR injury include intracellular calcium overload, inflammatory response, oxidative stress, mitochondrial dysfunction, and autophagy [2]. In 1972, Shen et al. [3] first discovered that transient coronary artery occlusion in dogs led to a rapid accumulation of intracellular calcium ions upon reperfusion and introduced the concept of “calcium overload”. Increasing evidence suggests that calcium overload is not only a major trigger of IR injury but also interacts with other mechanisms such as inflammation and oxidative stress, making it a critical focus in research on protective mechanisms against IR injury.

Calcium overload-induced mitochondrial dysfunction is a key mechanism leading to cell death during IR injury. Under conditions of calcium overload, mitochondria take up excessive calcium ions, resulting in mitochondrial calcium overload [4]. This triggers the opening of the mitochondrial permeability transition pore (mPTP), increasing mitochondrial membrane permeability and releasing pro-apoptotic factors such as cytochrome c into the cytosol, thereby activating caspase-9 and caspase-3, which induce apoptosis and further exacerbate myocardial injury [5].

Thus, mitochondria are considered a key therapeutic target for preventing cardiac IR injury. Several promising mitochondrial-targeted drugs are under development to counteract mitochondrial metabolic and morphological changes during IR injury [6, 7]. Promoting mitochondrial fusion and inhibiting mitochondrial fragmentation, while maintaining mitochondrial homeostasis, can help mitigate IR injury. Key regulators such as dynamin-related protein 1 (DRP-1) and mitofusin 2 (MFN2) play crucial roles, and targeting these pathways has shown potential for reducing necrosis and apoptosis by preserving mitochondrial integrity [8, 9]. Furthermore, calcium overload during IR injury is closely linked to interactions between mitochondria and the endoplasmic reticulum (ER), mediated through mitochondria-associated ER membranes (MAM). Dysregulated MAM contacts can disrupt cellular homeostasis, emphasizing the need for effective strategies to maintain these interactions [10, 11].

Despite advancements in understanding these mechanisms, traditional drugs targeting IR injury often lack specificity or show limited efficacy in improving mitochondrial dysfunction and calcium overload. For instance, some drugs focus on ROS scavenging or anti-inflammatory effects [12, 13], while others improve mitochondrial dynamics but fail to address the interactions between mitochondria and the ER, as well as the calcium homeostasis between them. This highlights the urgent need for novel therapies that can precisely target these interconnected pathways, providing comprehensive protection against IR injury.

Flavaglines are a class of natural compounds extracted from herbal plants that exhibit potent anti-cancer effects with no toxicity to healthy tissues [14]. Studies have shown that flavaglines not only exhibit selective toxicity against cancer cells but also promote the survival of cardiomyocytes under the adverse effects of chemotherapeutic drugs [15]. Our previous studies have found that Flavagline3 (FL3) promotes mitochondrial fusion dynamics, leading to more elongated mitochondrial structures [16]. We hypothesize that FL3 can target the balance between mitochondrial fusion and fission in cardiomyocytes, providing therapeutic benefits in myocardial IR injury.

## Results

### Flavagline3 Mitigates Acute Myocardial Apoptosis Induced by Ischemia-Reperfusion Injury in the Heart

**Fig. 1:**
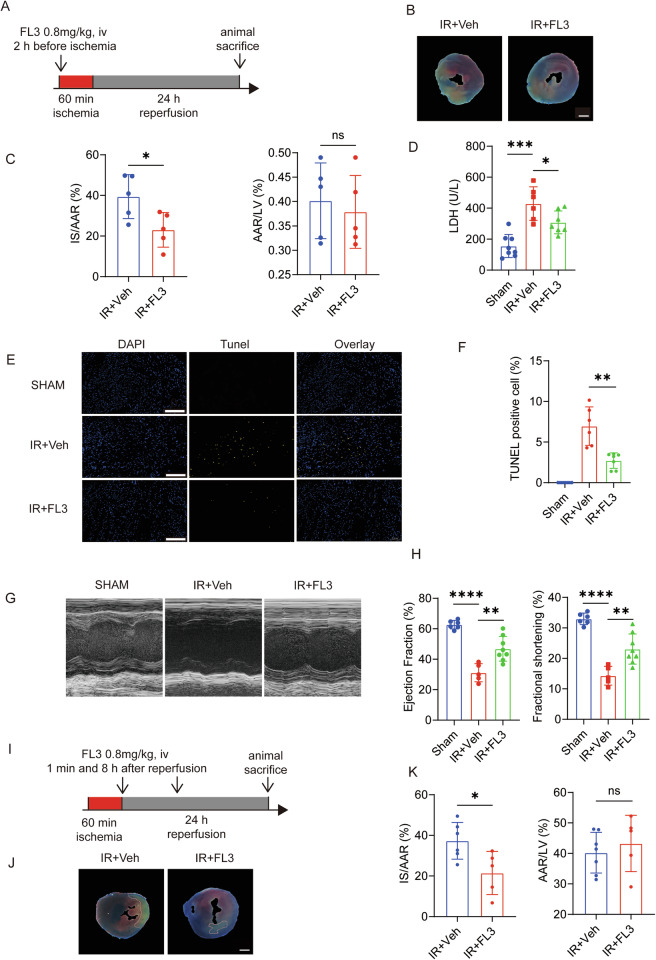
Flavagline3 (FL3) Mitigates Myocardial Apoptosis Induced by Ischemia-Reperfusion (IR) Injury in the Heart. **A** Experimental Design scheme: Mice were pretreated with a 0.8 mg/kg concentration of FL3 (IR + FL3 group) or DMSO (IR+Veh group) for two hours, and then both were subject to acute IR treatment (60 min ischemia followed by 24 hours reperfusion), all IR-treated animals were compared to the sham operation group. **B** Representative images of Evan’s blue and TTC double staining. **C** The quantitative data using images from panel B for infarct size (IF) (Left) and area at risk (AAR) (Right) (*n* = 5; scale bar: 1 mm). **D** The serum lactate dehydrogenase (LDH) concentration of sham operation group, IR+Veh group, and IR + FL3 group (*n* > 6). **E**, **F** The representative images (**E**) and statistical analysis data (**F**) of cardiac cell death indexed by TUNEL-positive cells (*n* = 8; scale bar: 0.2 mm). **G**, **H** Left ventricular ejection fraction (EF) and fractional shortening (FS) were evaluated by echocardiography (*n* = 6). **I** Experimental design scheme: Mice with acute IR injury (60 min ischemia followed by 24 h reperfusion) were divided into sham operation group, IR+Veh group, and IR + FL3 posttreatment group (administered after reperfusion onset). **J** Representative images of Evan’s blue and TTC double staining following posttreatment condition. **K** The quantitative data using images from panel J for infarct size (IF) (Left) and area at risk (AAR) (Right) (*n* = 5; scale bar: 1 mm). Data are presented as means ± SD. *P < 0.05, **P < 0.01, ***P < 0.001, ****P < 0.0001, ns, non-significant.

To determine whether Flavagline3 (FL3) can alleviate acute cardiac dysfunction and the extent of myocardial infarction induced by ischemia-reperfusion (IR), we first assessed the extent of myocardial infarction on the first day after IR modeling with FL3 pretreatment conditions (Fig. 1A). We found that FL3 reduced the infarct area caused by IR injury in the heart (Fig. 1B, C). We also measured the serum marker of myocardial injury, lactate dehydrogenase (LDH), and found that FL3 reduced the elevation of LDH by IR injury (Fig. 1D). Additionally, the proportion of terminal deoxynucleotidyl transferase dUTP nick-end labeling (TUNEL)-positive cells in the FL3 pretreated group (IR + FL3 group) was lower than that in the dimethyl sulfoxide (DMSO) treated group after IR injury (IR+Veh group), indicating that FL3 mitigated myocardial apoptosis induced by IR injury (Fig. 1E, F). Furthermore, echocardiography showed that IR + FL3 group had increased left ventricular ejection fraction (EF) and increased left ventricular fractional shortening (FS) compared to the IR+Veh group (Fig. 1G, H). Taken together, our data highlighted that FL3 pretreatment elevates the damage caused by IR. To better align with clinical application scenarios, we also assessed mice with post-reperfusion administration and found that FL3 could also significantly reduce the infarct area caused by IR injury (Fig. 1I–K).

### FL3 Attenuates Chronic Heart Failure and Cardiac Fibrosis Induced by IR Injury

**Fig. 2:**
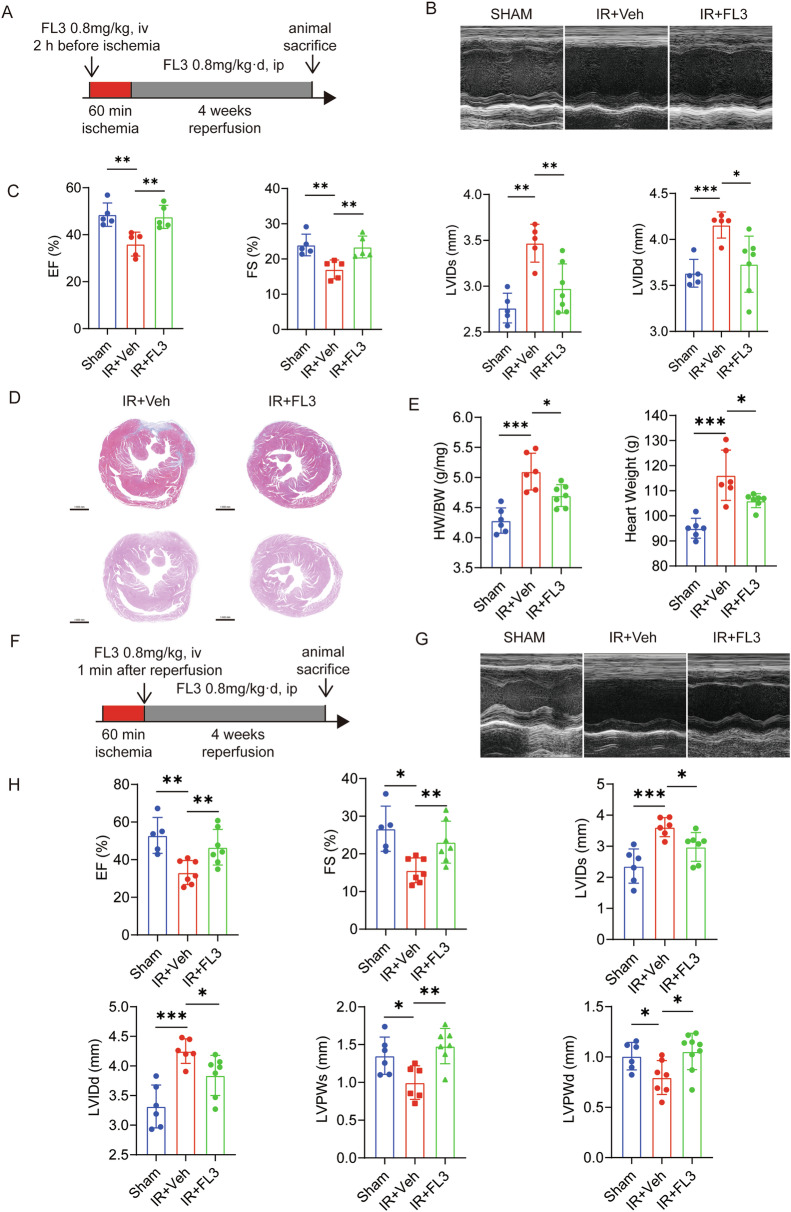
FL3 Attenuates Heart Failure and Cardiac Fibrosis Induced by IR Injury. **A** Experimental design scheme: Mice were subjected to chronic IR injury (60 minutes ischemia followed by 4 weeks reperfusion). The animals were divided into three groups: sham operation group, IR+Veh group, and IR + FL3 treatment group (administered FL3 at 2 h before ischemia, followed by daily intraperitoneal injection of 0.8 mg/kg·d). **B**, **C** EF and FS were evaluated by echocardiography (*n* = 5 per group). **D, E** Myocardial infarction severity was assessed by Masson’s trichrome staining (Top) and HE staining (Bottom), as well as the heart weight to body weight ratio (HW/BW) and heart weight (n = 5 per group). These data were obtained from mice with chronic IR injury treated with either DMSO or FL3 (0.8 mg/kg·d), following the protocol outlined in A. **F** Mice subjected to chronic IR injury (60 min ischemia followed by 4 weeks reperfusion) were treated post-reperfusion with either DMSO (IR+Veh) or FL3 (administered at 1 minute and 4 h post-reperfusion, followed by daily intraperitoneal injections of 0.8 mg/kg·d). **G**, **H** EF and FS were measured by echocardiography (*n* = 6 [sham], *n* = 6 [IR+Veh group], and *n* = 7 [IR + FL3 group]). Data are presented as mean ± SD. *P < 0.05, **P < 0.01, ***P < 0.001.

To determine whether FL3 can alleviate chronic heart failure and cardiac fibrosis induced by IR, we assessed the cardiac function of mice continuously injected intraperitoneally with DMSO or FL3 for 4 weeks using echocardiography in the 4th week after IR modeling. We found that pretreatment with FL3 (Fig. 2A) followed by continuous administration of FL3 for 4 weeks alleviated left ventricular systolic dysfunction. Echocardiography showed that, compared to the IR+Veh group, mice continuously treated with FL3 had increased EF, increased FS, decreased left ventricular internal dimension in end-systole (LVIDs), and decreased left ventricular internal dimension in end-diastole (LVIDd) (Fig. 2B, C). This indicated that FL3 alleviated chronic heart dysfunction caused by IR injury. Masson’s trichrome and Hematoxylin and eosin (HE) staining indicated that FL3 reduced chronic cardiac fibrosis following IR injury (Fig. 2D). Furthermore, compared to the IR+Veh group, the IR + FL3 group exhibited a lower heart weight and heart weight/body weight ratio (Fig. 2E), indicating that FL3 mitigated IR-induced cardiac remodeling. Similarly, we found that mice continuously treated with FL3 had increased EF, increased FS, decreased LVIDs, and decreased LVIDd in the FL3 post-treatment group compared to those of the IR+Veh group (Fig. 2F–H). Moreover, the IR + FL3 group showed increased left ventricular posterior wall thickness in end-systole (LVPWs) and end-diastole (LVPWd). The above results suggested that continuous administration of FL3 can effectively mitigate heart dysfunction and cardiac remodeling following IR injury.

### FL3 Alleviates Hypoxia/Reoxygenation Induced Cardiomyocyte Apoptosis

**Fig. 3:**
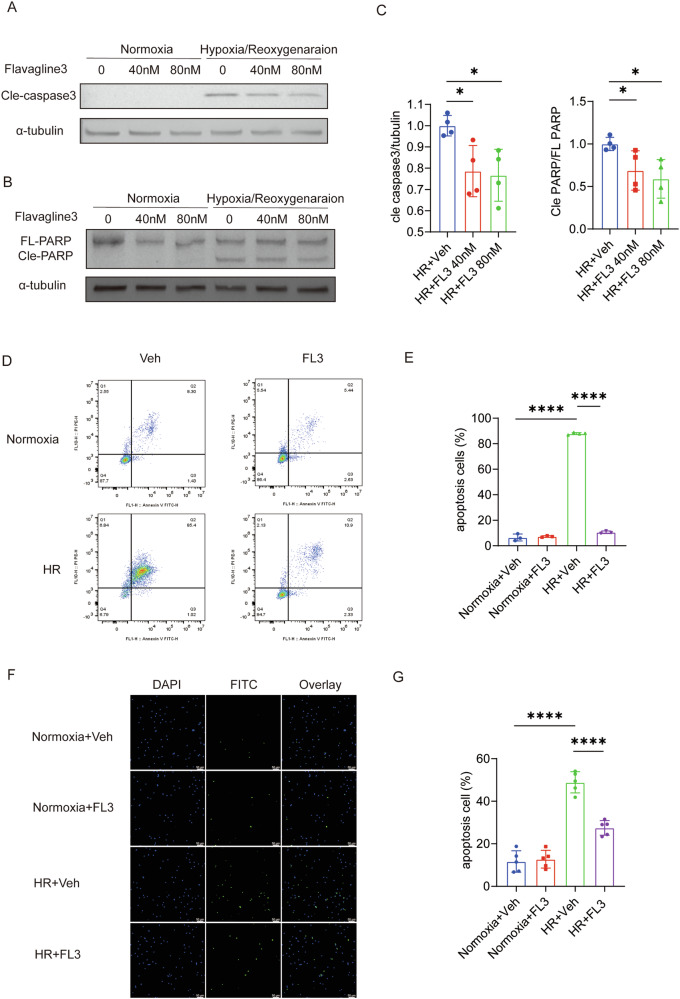
FL3 Alleviates Hypoxia/Reoxygenation Induced Cardiomyocyte Apoptosis. **A**–**C** HL-1 cells were subjected to hypoxia/reoxygenation (HR) (12 h of hypoxia followed by 2 h of reoxygenation) with or without treatment with varying concentrations of FL3 (40 and 80 nM). Cleaved caspase-3 (H, *n* = 4) and cleaved PARP levels were evaluated using Western blots and statistical analysis. **D**, **E** The level of apoptosis was assessed by flow cytometry. **F** Representative images of neonatal rat ventricular myocytes (NRVMs) exposed to HR (24 h of hypoxia followed by 2 h of reoxygenation) with or without FL3 treatment (80 nM). **G** The proportion of TUNEL-positive cells in the culture medium was quantified. Data are expressed as mean ± SD. *P < 0.05, ****P < 0.0001.

To further investigate the protective mechanism of FL3 on cardiomyocytes during IR injury, we constructed an in vitro model of hypoxia/reoxygenation (HR) injury in cardiomyocytes. The results showed that, following HR injury in HL-1 cells, the expression of cleaved-Poly (ADP-ribose) polymerase (PARP) and cleaved-caspase-3 was reduced in FL3-treated HL-1 cells compared to the control group (Fig. 3A–C). Additionally, flow cytometry analysis similarly confirmed that FL3 alleviated apoptosis induced by HR injury (Fig. 3D, E). Furthermore, in neonatal rat ventricular myocytes (NRVMs), the proportion of TUNEL-positive cells was reduced in the HR + FL3 group compared to the HR+Veh group (Fig. 3F, G), indicating that FL3 decreased apoptosis levels in NRVMs induced by HR.

### FL3 Increases Mitochondria-Associated Endoplasmic Reticulum Membranes and Promotes Mitochondrial Fusion in the Myocardial IR Injury

**Fig. 4:**
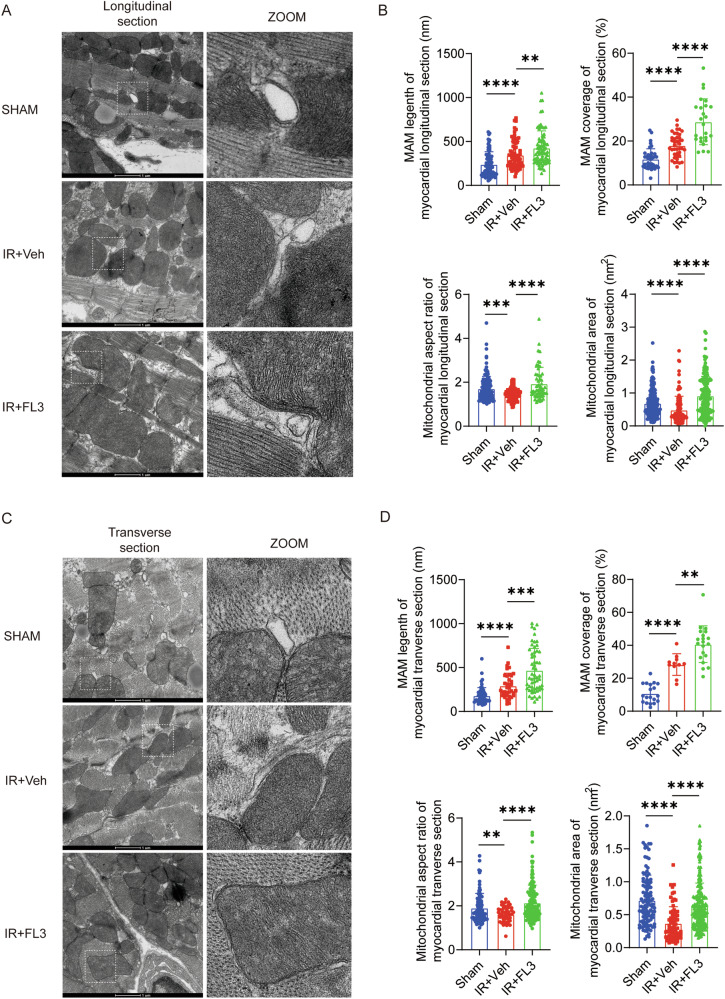
FL3 Increases Mitochondria-Associated Endoplasmic Reticulum Membranes and Promotes Mitochondrial Fusion in the Myocardial IR Injury. **A** Transmission electron microscopy (TEM) images (longitudinal section) of myocardial tissues from the sham operation, IR+Veh group, and IR + FL3 group were obtained to evaluate mitochondrial morphology. **B** Quantitative analysis of TEM images from A was performed, including measurements of mitochondria-associated endoplasmic reticulum membranes (MAM) length per mitochondrion (nanometers), percentage of endoplasmic reticulum coverage around mitochondria, mitochondrial aspect ratio, and mitochondrial area (nm²). **C** TEM images (transverse section) of myocardial tissues from the sham operation, IR+Veh group, and IR + FL3 group were obtained to evaluate mitochondrial morphology. **D** Quantitative analysis of TEM images from A was performed, including measurements of MAM length per mitochondrion (nanometers), percentage of endoplasmic reticulum coverage around mitochondria, mitochondrial aspect ratio, and mitochondrial area (nm²). Data are presented as means ± SD. **P < 0.01, ***P < 0.001, ****P < 0.0001.

To investigate the impact of FL3 on mitochondrial morphology and mitochondrial-endoplasmic reticulum (ER) interactions in myocardial tissues following IR injury, we utilized transmission electron microscopy. We aimed to determine whether FL3 could mitigate mitochondrial fragmentation and enhance mitochondria-associated ER membranes (MAM) formation, which are critical for cardiomyocyte survival during IR injury. Our findings demonstrated that mitochondrial fragmentation was significantly more pronounced in the IR injury group compared to the sham group. In the IR+Veh group, both the mitochondrial aspect ratio and area were significantly reduced compared to the sham group; however, these parameters were restored in the IR + FL3 group. This indicated that FL3 effectively reversed the mitochondrial fission induced by IR injury. Moreover, we observed that the interaction between mitochondria and the ER was significantly increased in the IR+Veh group compared to the sham group (Fig. 4A). This was further supported by quantitative analyses revealing enhanced MAM coverage and length in longitudinal sections of myocardial tissue (Fig. 4B). Following FL3 treatment, both MAM coverage and length further increased compared to the IR+Veh group, suggesting that FL3 enhanced mitochondrial-ER interactions. Transverse section analyses corroborated these results (Fig. 4C, D). In conclusion, our data indicated that FL3 not only reversed mitochondrial fragmentation following IR injury but also promoted the formation of MAM, thereby potentially enhancing cellular resilience during oxidative stress.

### FL3 Promotes Mitochondrial Fusion Through MFN1 During HR

**Fig. 5:**
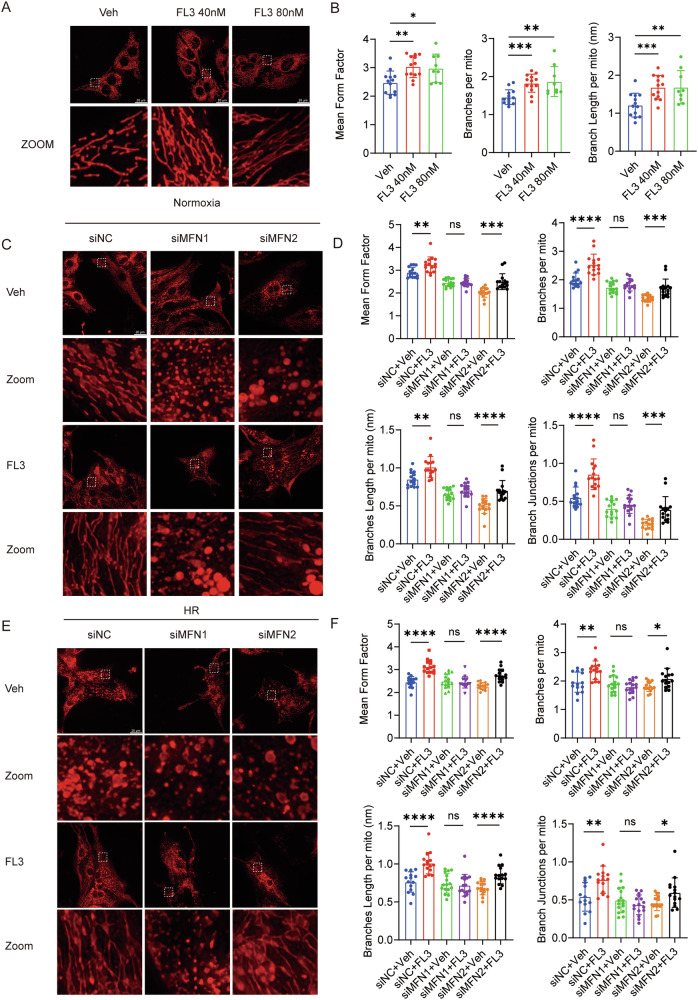
FL3 Promotes Mitochondrial Fusion Through MFN1 During HR. **A** Representative images of HL1 cells subjected to HR, stained with MitoTracker to assess mitochondrial fusion after FL3 treatment. **B** Quantitative parameters evaluated from the images, including mean form factor, average branch per mitochondrion, average branch length per mitochondrion (nanometers). **C** Representative images of NRVM cells under MFN1 and MFN2 knockdown conditions, stained with MitoTracker following FL3 treatment to assess mitochondrial fusion. **D** Quantitative parameters evaluated from **C**, including mean form factor, branches per mitochondrion, average branch length per mitochondrion (nanometers), and branch junctions per mitochondrion. **E** Representative images of NRVMs under MFN1 and MFN2 knockdown conditions, stained with MitoTracker after HR and FL3 treatment. **F** Quantitative parameters evaluated from (**E**), including mean form factor, branches per mitochondrion, average branch length per mitochondrion (nanometers), and branch junctions per mitochondrion. All quantitative data are presented as mean ± SD. *P < 0.05, **P < 0.01, ***P < 0.001, ****P < 0.0001, ns, non-significant.

A possible explanation for the beneficial effect of FL3 could be due to its connection to mitochondria. To determine whether FL3 affects mitochondrial morphology in cardiomyocytes and to explore whether this effect is related to FL3’s protective mechanism in HR injury, we treated HL-1 cardiomyocytes with FL3 and found that FL3 promoted the elongation and fusion of mitochondrial structures (Fig. 5A, B). In NRVMs, we found that FL3 can effectively restore mitochondrial morphology under HR (Fig. 5C, D). Mitofusin (MFN) is localized to the outer mitochondrial membrane (OMM) and mediates its fusion [17]. In higher eukaryotes, there are two functionally overlapping MFNs, namely MFN1 and MFN2 [18]. Overexpression or knockout mutations of these proteins can both alter mitochondrial morphology in cell lines [19, 20]. Notably, we found that FL3 upregulated mRNA levels of both MFN1 and MFN2 while leaving OPA1 expression unchanged (Figure S1A). Knocking down (KD) either MFN (MFN1 KD or MFN2 KD) led to mitochondrial fragmentation in NRVMs, but we found that FL3 can rapidly rescue mitochondrial fragmentation caused by HR in MFN2 KD NRVMs (Fig. 5C, D). However, it failed to work in MFN1 KD NRVMs (Fig. 5E, F). We obtained the same results in HeLa cells (Figure S2). Taken together, FL3 was effective in promoting mitochondrial fusion only in an MFN1-dependent manner.

### FL3 Maintains Calcium Homeostasis and Protects Mitochondrial Function

**Fig. 6:**
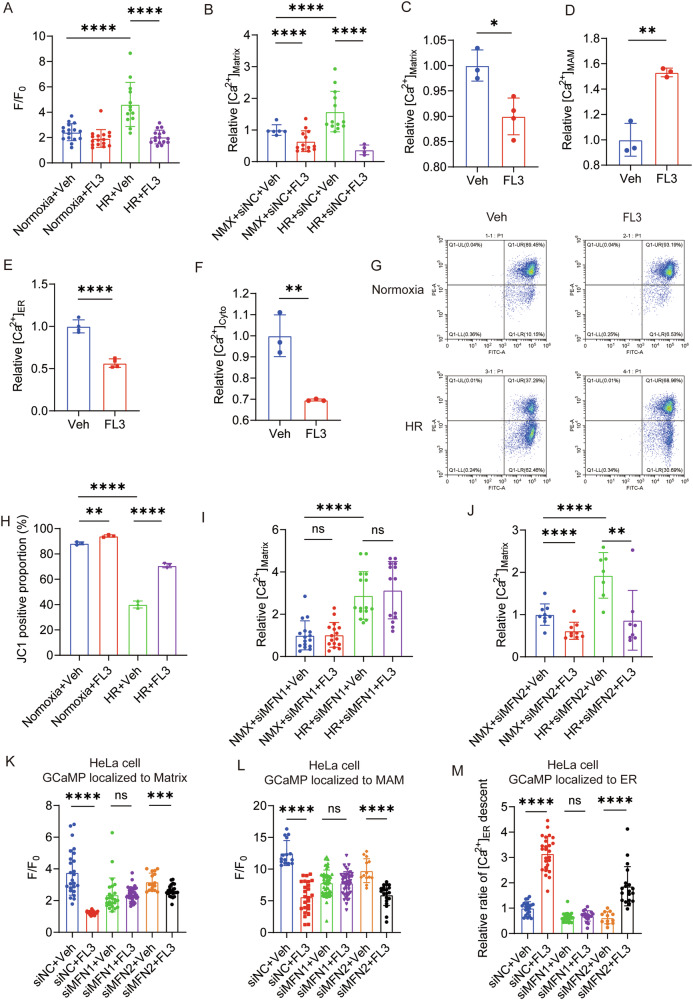
FL3 Maintains Calcium Homeostasis and Protects Mitochondrial Function. **A** Peak calcium ion flux in the mitochondrial matrix of NRVMs following histamine stimulation, FL3 treatment, and HR. **B** Mitochondrial matrix calcium ion concentrations in NRVMs under FL3 treatment and HR. **C**–**F** Calcium ion concentrations in various subcellular compartments following FL3 treatment: (**C**) mitochondrial matrix, (**D**) MAM, (**E**) endoplasmic reticulum (ER), and (**F**) cytoplasm (Cyto). **G**, **H** JC-1 staining was used to assess mitochondrial membrane potential (*n* = 3), (**G**) Representative images, (**H**) Quantified JC-1 red/green fluorescence ratio. **I**, **J** Mitochondrial matrix calcium ion concentrations in NRVMs under FL3 treatment and HR analyzed with MFN1 knockdown (**I**), and with MFN2 knockdown (**J**), respectively. **K**, **L** Peak calcium ion flux in both the mitochondrial matrix and MAM under FL3 treatment in response to histamine stimulation: (**K**) mitochondrial matrix, (**L**) MAM. **M** The relative maximum rate of calcium release from the ER in HeLa cells following FL3 treatment in response to histamine stimulation. All quantitative data are presented as mean ± SD. NMX, normoxia; F/F_0_, peak-to-baseline ratio; *P < 0.05, **P < 0.01, ***P < 0.001, ****P < 0.0001, ns, non-significant.

During IR injury, mitochondrial calcium overload is one of the key mechanisms leading to cardiomyocyte loss [21, 22]. Our results indicated that FL3 significantly increased mitochondrial fusion and the volume of individual mitochondria (Fig. 5A, B). We hypothesized that these morphological changes may enhance FL3’s ability to modulate mitochondrial calcium ion sequestration, thereby buffering calcium overload. Therefore, we further investigated the effects of FL3 on calcium homeostasis in cardiomyocytes. Under HR, the peak calcium ion flux in the mitochondrial matrix of NRVMs increased, which was attenuated by FL3 treatment (Fig. 6 A). This suggested that FL3 alleviated HR-induced mitochondrial calcium overload. We subsequently measured the calcium ion concentration in the mitochondrial matrix of NRVMs after HR injury. The results revealed that HR increased the calcium ion concentration in the mitochondrial matrix. FL3 not only reduced mitochondrial matrix calcium under normoxic conditions but also lowered it under HR (Fig. 6B). These findings suggested that FL3 effectively mitigated mitochondrial calcium overload during HR, enhancing the mitochondrial capacity for calcium ion sequestration and potentially protecting cardiomyocytes from cell death.

Furthermore, FL3 treatment reduced the calcium ion concentration in the mitochondrial matrix of HeLa cells, increased MAM levels, and decreased calcium levels in both the ER and cytosol (Fig. 6C–F). We also assessed the impact of FL3 on mitochondrial membrane potential using JC-1 staining, finding that FL3 treatment significantly reduced the mitochondrial membrane potential (MMP) (Fig. 6G, H). This indicated that FL3 affected mitochondrial function under HR conditions. We then investigated the roles of MFN1 and MFN2 in FL3’s regulation of calcium homeostasis. This effect remained significant under MFN2 knockdown conditions but was ineffective in MFN1 knockdown NRVMs (Fig. 6I, J). Additionally, after FL3 treatment, the peak calcium ion flux in the mitochondrial matrix of HeLa cells was reduced under histamine stimulation, and the calcium ion peak in the MAM region also decreased (Fig. 6K, L). In MFN2 KD HeLa cells, the peak calcium ion flux in the mitochondrial matrix and MAM still decreased following FL3 treatment under histamine stimulation, but this was not observed in MFN1 KD HeLa cells (Fig. 6K, L). Furthermore, we found that the maximum rate of calcium release from the ER decreased after FL3 treatment under histamine stimulation, while the effect was attenuated by MFN1 KD but not by MFN2 KD (Fig. 6M). To further delineate the mitochondrial mechanism of FL3 as a PHB ligand, we systematically investigated potential crosstalk between MFN1 and the PHB-STAT3 pathway. Intriguingly, while FL3 altered the nuclear-to-cytoplasmic ratio of PHBs, its inhibition of STAT3 phosphorylation and maintenance of cytoplasmic PHB levels remained unaffected by MFN1 KD condition (Figure S1B). The distinct calcium dynamics in MFN1 KD cells identified MFN1 as a key mediator of FL3's cardioprotection, nominating it as a potential therapeutic target for cardiac injury.

## Discussion

Myocardial ischemia-reperfusion (IR) injury is a significant cause of cardiac dysfunction following acute myocardial infarction, and effective clinical treatments against IR injury remain limited. This study elucidates the potential applications of Flavagline3 (FL3) in cardiology, particularly its value as a cardioprotective agent. The results indicate that FL3 offers significant protection against myocardial IR injury. Experimental findings demonstrate that FL3 effectively reduces infarct size, improves cardiac function, and inhibits cardiomyocyte apoptosis. Echocardiographic assessments show that all measured parameters in the FL3 treatment group are significantly better than those in the control group, indicating that FL3 can effectively enhance cardiac contractility. Moreover, the application of FL3 in chronic stages also yields positive outcomes, as long-term treatment alleviates cardiac dysfunction, reduces the degree of cardiac fibrosis, and mitigates the incidence of cardiac remodeling. Clinically, this may contribute to lowering mortality rates following cardiac reperfusion injury and improving patients’ quality of life. These findings suggest that FL3 could play a beneficial role in reducing mortality after cardiac reperfusion injury and enhancing patient quality of life, potentially offering new directions for the treatment of cardiovascular diseases.

FL3 demonstrates significant protective effects in a hypoxia/reoxygenation (HR) model, particularly in maintaining mitochondrial morphology and function. Studies indicate that FL3 effectively inhibits mitochondrial fission and promotes fusion. This protection is critical for sustaining cardiomyocyte function, especially under HR-induced stress conditions, where mitochondria typically undergo substantial morphological changes, leading to dysfunction and cell death. Further investigations reveal that the promotion of mitochondrial fusion by FL3 is mediated by a mechanism dependent on mitofusin1 (MFN1). MFN1/2 are key proteins on the outer mitochondrial membrane responsible for mediating mitochondrial fusion processes [19]. Previous research has shown that drugs can enhance endogenous MFN1 to promote mitochondrial fusion and protect the heart from IR injury [23].

Notably, as a high-affinity ligand of PHB, FL3 modulates mitochondrial signaling pathways beyond its previously established roles in cardioprotection [24–26]. Prior studies have demonstrated that FL3 exerts cardioprotective effects by relocalizing PHB to mitochondria and suppressing STAT3 phosphorylation [15]. Building on these findings, we investigated potential crosstalk between PHB-STAT3 signaling and MFN1-dependent mechanisms under MFN1 knockdown (KD) condition. Intriguingly, while FL3 reduced nuclear PHB accumulation in MFN1 KD condition, its suppression of STAT3 phosphorylation remained consistent regardless of MFN1 status. Critically, cytoplasmic PHB levels and STAT3 inactivation were unaffected by MFN1 depletion, indicating that these effects operate independently of FL3’s MFN1-dependent mitochondrial fusion pathway. Our data unequivocally identify MFN1 as the central mediator of FL3’s cardioprotection against IR injury, primarily through promoting mitochondrial fusion and stabilizing calcium homeostasis. In contrast, the PHB-STAT3 pathway appears peripherally related to FL3’s primary mechanism in this context, as cytoplasmic PHB levels showed no significant changes. However, the biological implications of FL3-driven nuclear PHB reduction under MFN1 KD condition—a paradoxical reversal of its normal nuclear accumulation—warrant further investigation. We speculate that FL3’s MFN1-dependent effects (fusion/calcium homeostasis regulation) and its PHB-STAT3 modulation may engage in context-specific crosstalk, though this hypothesis requires rigorous validation.

In the context of myocardial IR injury, the endoplasmic reticulum (ER) serves as a calcium reservoir [27], and calcium overload is widely recognized as a key trigger of cardiac damage [28]. Therefore, interventions targeting the physical and functional coupling between mitochondria and the ER may effectively improve IR injury. Compared to existing cardioprotective drugs, FL3 exhibits a unique mechanism in regulating calcium homeostasis. Our research indicates that FL3 significantly increases the coverage area and length of the mitochondria-associated ER membranes (MAM), enhancing the interaction between mitochondria and the ER. Upon FL3 treatment, calcium redistributes within the cell, resulting in decreased concentrations in the mitochondrial matrix, ER, and cytoplasm, while increasing levels in the MAM region. Interestingly, knocking down MFN2, which typically reduces Mitochondrial-ER interactions, did not diminish the effect of FL3 on mitochondrial matrix calcium levels. These findings suggest that although FL3 clearly influences Mitochondrial-ER interactions, its impact on calcium levels in the mitochondrial matrix may occur through mechanisms independent of these interactions.

FL3, as a novel cardioprotective agent, has been validated for its safety in multiple studies. Research indicates that long-term administration of FL3 does not result in significant adverse effects on healthy tissues or other organs. This finding is critical for its clinical application as a therapeutic agent. The favorable safety profile of FL3 provides a robust foundation for its use in treating cardiovascular diseases, thereby enhancing clinicians’ confidence in its application. In terms of clinical translation, FL3 shows promising potential, particularly in the treatment of acute myocardial injury, where its efficacy and safety profile support its candidacy as a therapeutic option.

## Supplementary information

Supplemental Figure legends

Figure S1

Figure S2

Figure S3

Figure S4

Figure S5

Full length WB of figure3A

Full length WB of figure3B

Full length WB partI of figureS1-B

Full length WB partII of figureS1-B

Full length WB of figureS5- (A+B) -1

Full length WB of figureS5- (A+B) -2

Full length WB of figureS5- (A+B) -3

Full length WB of figureS5-C+D

Full length WB of figureS5-E

methods

## Data Availability

All data and materials are available as described in the Nature Academic Journals Reporting Checklist (Supplementary File). Additional data or materials are available from the corresponding author upon reasonable request.
