# Supplementary material for: FL3 mitigates cardiac ischemia-reperfusion injury by promoting mitochondrial fusion to restore calcium homeostasis
Source: Cell Death Discov. 2025 Jul 3;11:304. doi: 10.1038/s41420-025-02575-w (PMC12229567; doi:10.1038/s41420-025-02575-w)
Supplement: Supplementary file 1 — Supplemental Figure legends [file 41420_2025_2575_MOESM1_ESM.docx]

**List of supplemental figure legends**

**Supplemental Figure 1. FL3 regulates PHB subcellular localization and MFN1/2 transcription in cardiomyocytes.** A. qPCR analysis of MFN1, MFN2, and OPA1 mRNA levels in FL3-treated (80 nM, 24h) cardiomyocytes. B. Western blot analysis of nuclear and cytoplasmic fractions from HL-1 cardiomyocytes treated with FL3 (80 nM, 24h) under four conditions: siNC + Vehicle, siNC + FL3, siMFN1 + Vehicle, siMFN1 + FL3.

**Supplemental Figure 2. FL3 Promotes Mitochondrial Fusion Through MFN1 in HeLa Cell.**

A. Representative images of HeLa cells under MFN1 and MFN2 knockdown conditions, stained with MitoTracker following FL3 treatment to assess mitochondrial fusion (scale bar = 20 um). B. Quantitative parameters evaluated from A, including, branches per mitochondrion, average branch length per mitochondrion (nanometers), average branch length per branch (nanometers), and branch junctions per mitochondrion. All quantitative data are presented as mean ± SD. **P*<0.05, ***P*<0.01, ****P*<0.001, *****P*<0.0001.

**Supplemental Figure 3. FL3 Protects Mitochondrial Function under H/R**

A. Representative oxygen consumption rate (OCR) curves for cardiomyocytes treated with FL3 and subjected to H/R, illustrating changes in mitochondrial respiration over time. B-E. Quantification of specific OCR parameters, including basal respiration (B), ATP-linked respiration (C), maximal respiration (D), and spare respiratory capacity (E), shown as bar graphs for different treatment groups, highlighting FL3’s effects on mitochondrial respiration. F. Representative extracellular acidification rate (ECAR) curves depicting glycolytic function under HR conditions. G-I. Bar graphs of glycolytic parameters, including basal glycolysis (G), glycolytic capacity (H), and glycolytic reserve (I), demonstrating that FL3 treatment maintains ECAR levels following HR

**Supplemental Figure 4. FL3 treatment does not affect the phenotypes of the mice.**

A. Experimental design schematic showing intraperitoneal injection of either DMSO or FL3 in two groups of mice over four weeks. B. Body weight, heart-to-body weight ratio, and hematological parameters, including red blood cell count, white blood cell count, platelet count, and hemoglobin levels. C. HE staining of major organs, including the heart, kidney, lung, and liver, from both treatment groups (scale bar = 0.1mm).

**Supplemental Figure 5. Protein Expression Levels After MFN1 and MFN2 Knockdown**

A. Western blot validation of MFN1 and MFN2 expression in NRVMs after MFN1 knockdown. B. Western blot validation of MFN2 and MFN1 expression in NRVMs after MFN2 knockdown. C. Western blot validation of MFN1 expression in HeLa cells after MFN1 knockdown. D. Western blot validation of MFN2 expression in HeLa cells after MFN2 knockdown. E. Western blot analysis of MFN1 expression levels in HL-1 cardiomyocytes under HR conditions with and without FL3 treatment.

**Supplemental Figure 6. FL3 exerts robust cardioprotective effects through its ability to promote mitochondrial fusion, enhance mitochondrial-ER interactions, and maintain calcium homeostasis.**
