# Supplementary figures and images for: FL3 mitigates cardiac ischemia-reperfusion injury by promoting mitochondrial fusion to restore calcium homeostasis
Source: Cell Death Discov. 2025 Jul 3;11:304. doi: 10.1038/s41420-025-02575-w (PMC12229567; doi:10.1038/s41420-025-02575-w)

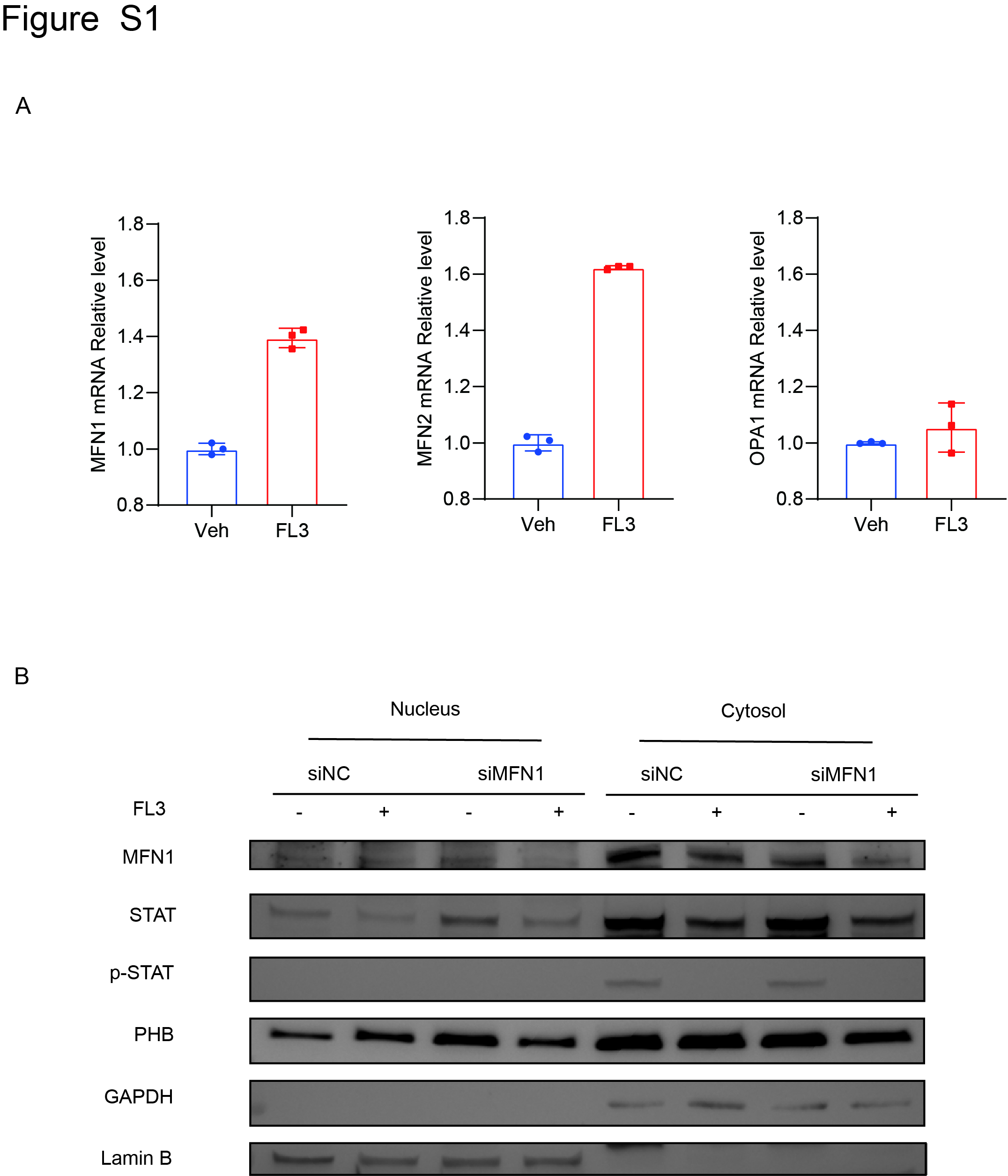

Supplement: Supplementary file 2 — Figure S1 [file 41420_2025_2575_MOESM2_ESM.tif]

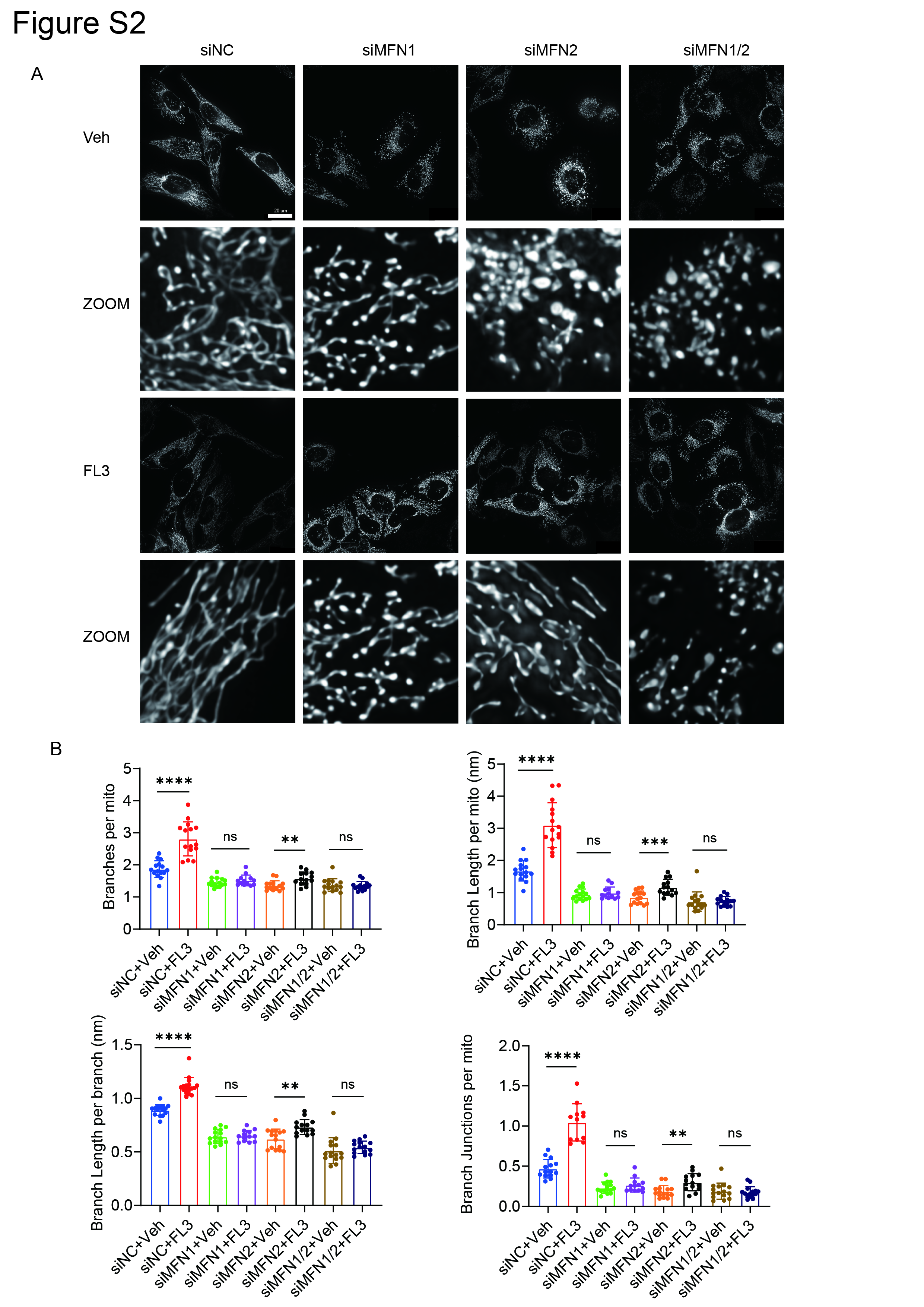

Supplement: Supplementary file 3 — Figure S2 [file 41420_2025_2575_MOESM3_ESM.tif]

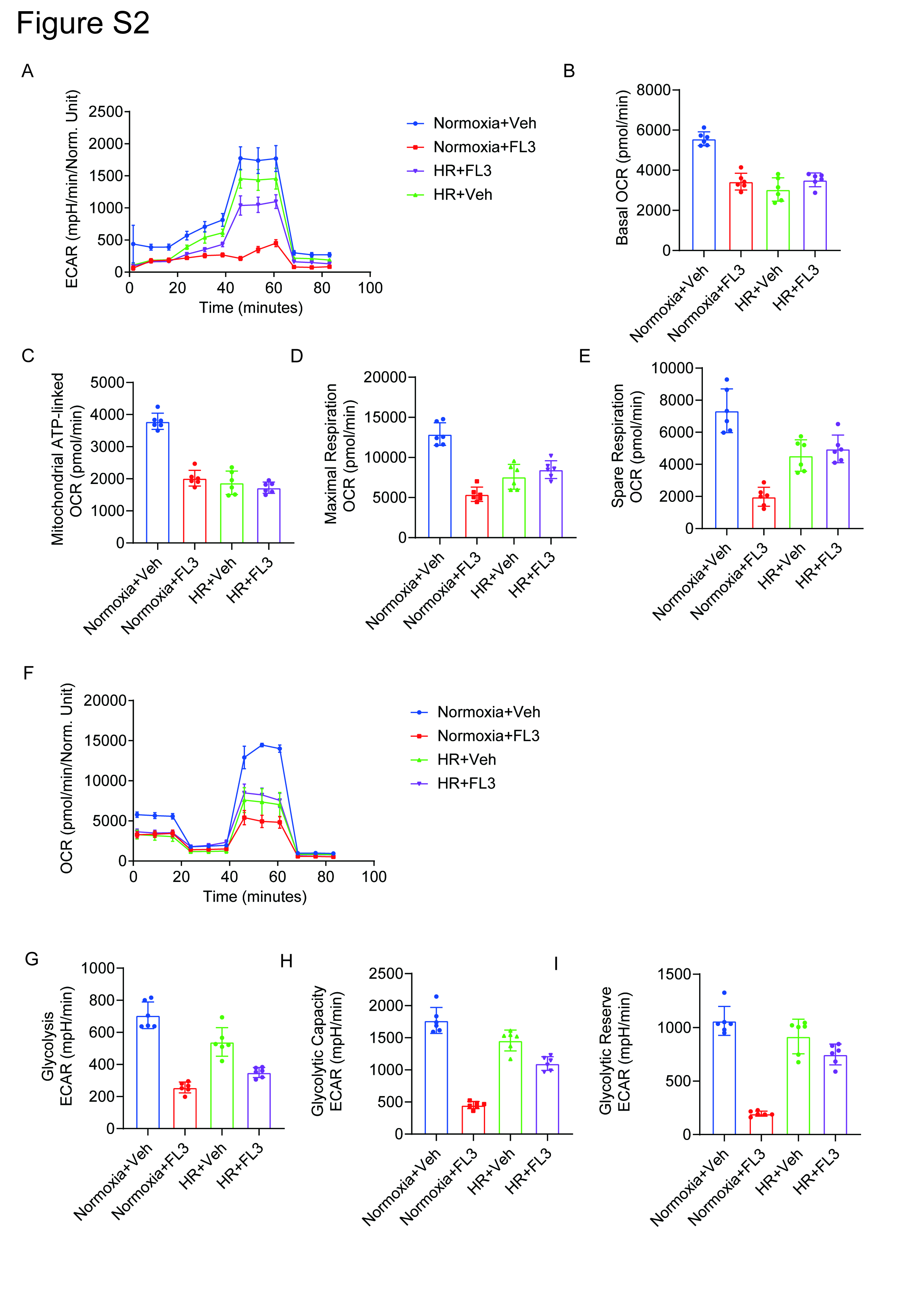

Supplement: Supplementary file 4 — Figure S3 [file 41420_2025_2575_MOESM4_ESM.tif]

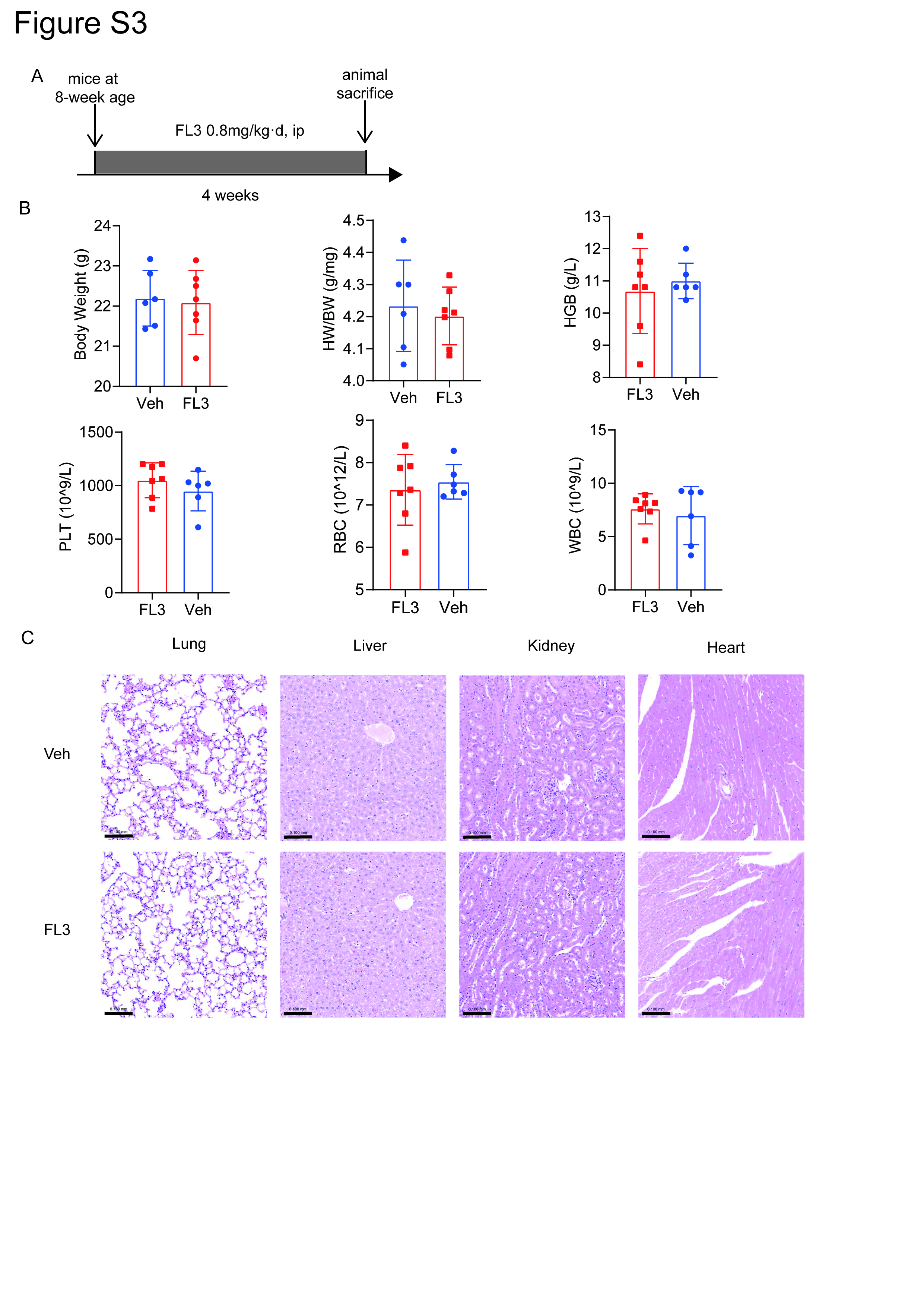

Supplement: Supplementary file 5 — Figure S4 [file 41420_2025_2575_MOESM5_ESM.tif]

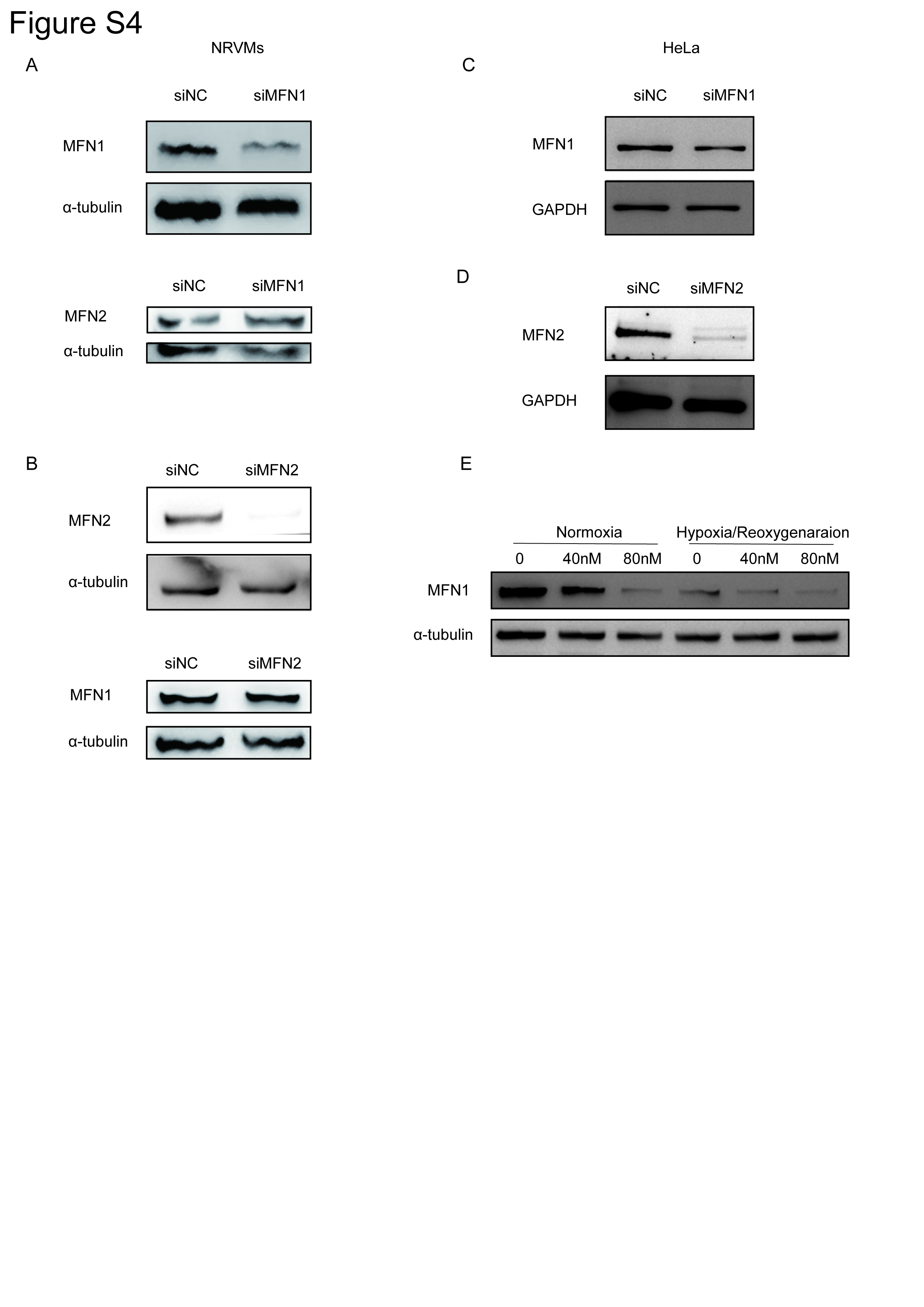

Supplement: Supplementary file 6 — Figure S5 [file 41420_2025_2575_MOESM6_ESM.tif]

| FL3 | NMX |      |      | HR  |      |      |
|-----|-----|------|------|-----|------|------|
|     | 0nM | 40nM | 80nM | 0nM | 40nM | 80nM |

PARP  
Cle-PARP

100KD

70KD

Tubulin

50KD

40KD

35KD

25KD

Cle-cas3

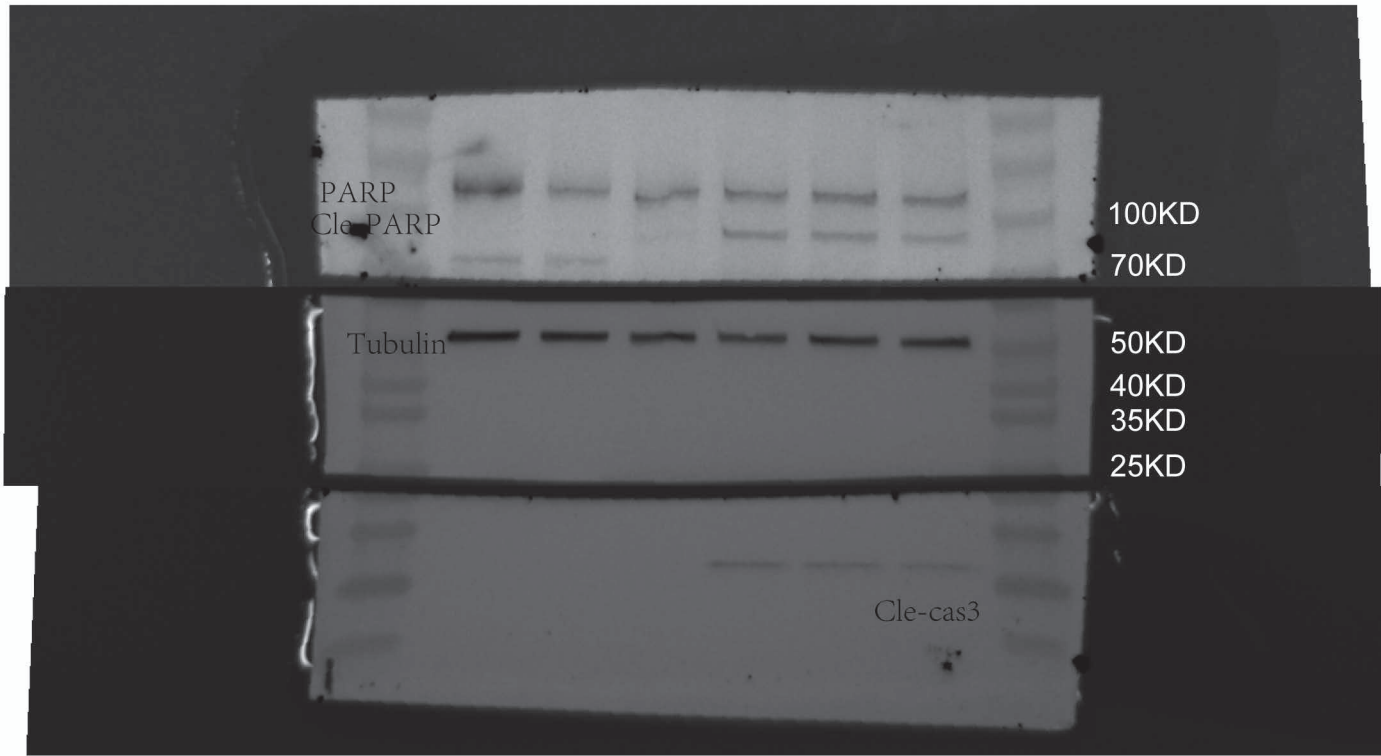

Supplement: Supplementary file 7 — Full length WB of figure3A [file 41420_2025_2575_MOESM7_ESM.pdf]

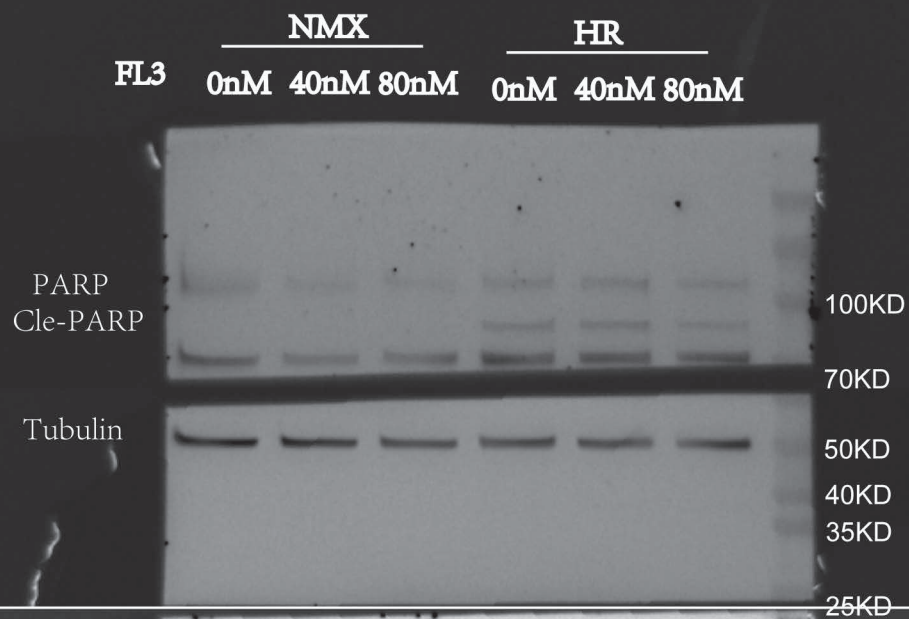

Cle-cas3

Supplement: Supplementary file 8 — Full length WB of figure3B [file 41420_2025_2575_MOESM8_ESM.pdf]

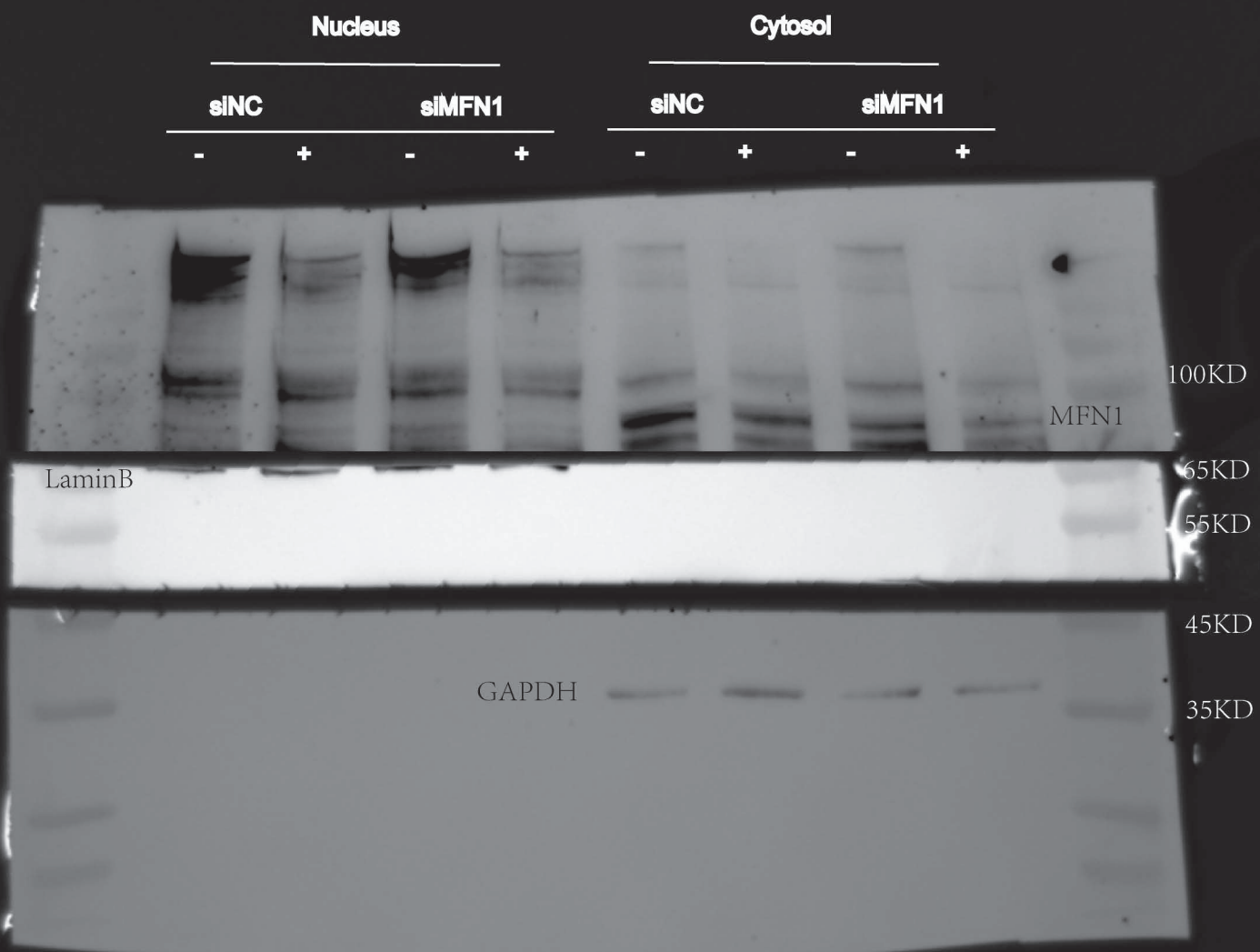

Supplement: Supplementary file 9 — Full length WB partI of figureS1-B [file 41420_2025_2575_MOESM9_ESM.pdf]

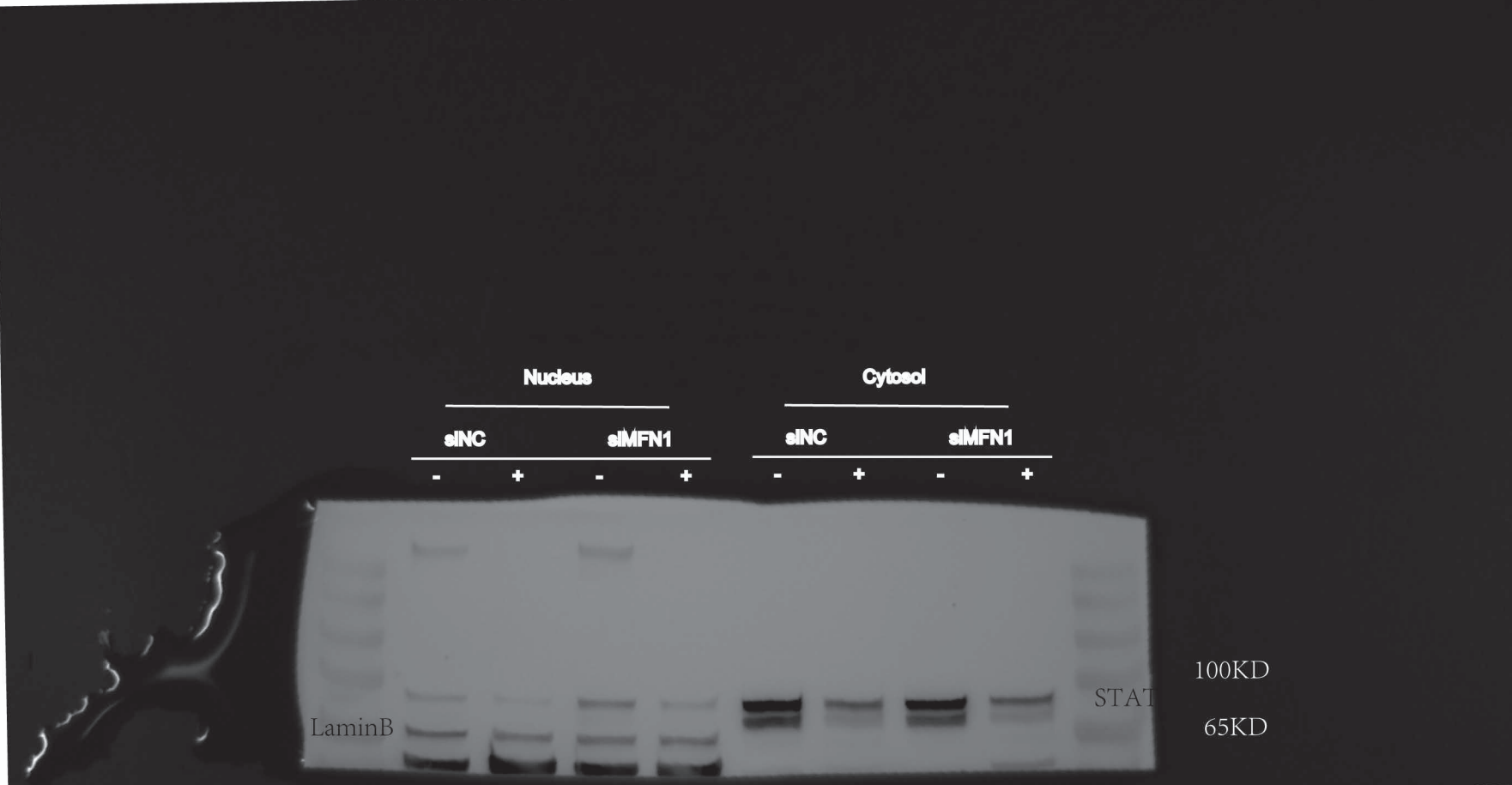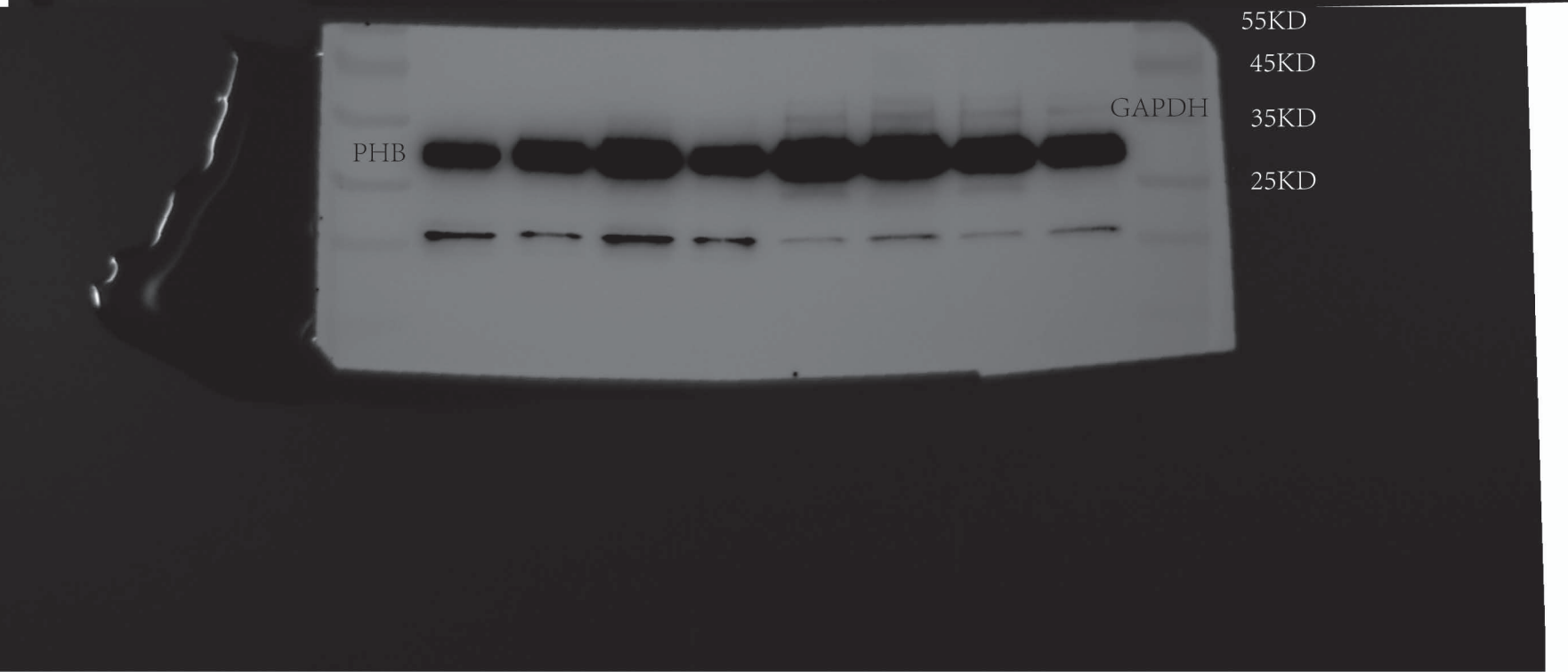

Supplement: Supplementary file 10 — Full length WB partII of figureS1-B [file 41420_2025_2575_MOESM10_ESM.pdf]

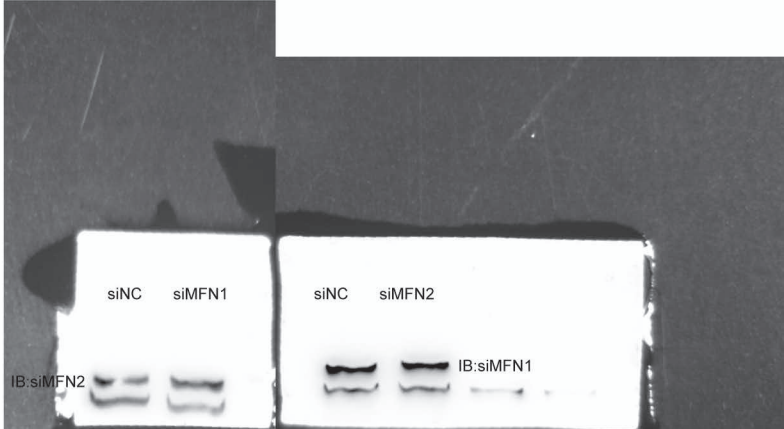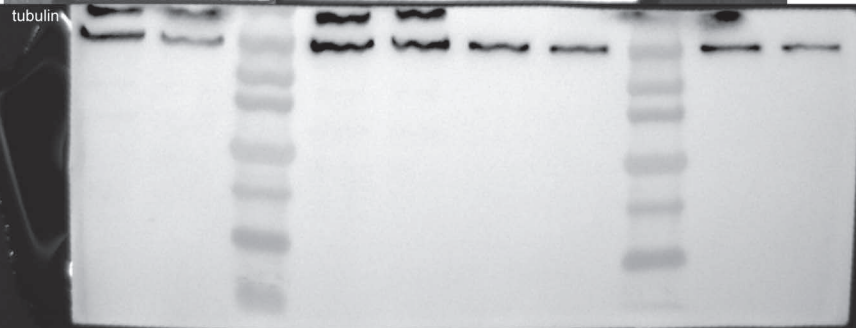

Supplement: Supplementary file 11 — Full length WB of figureS5- (A+B) -1 [file 41420_2025_2575_MOESM11_ESM.pdf]

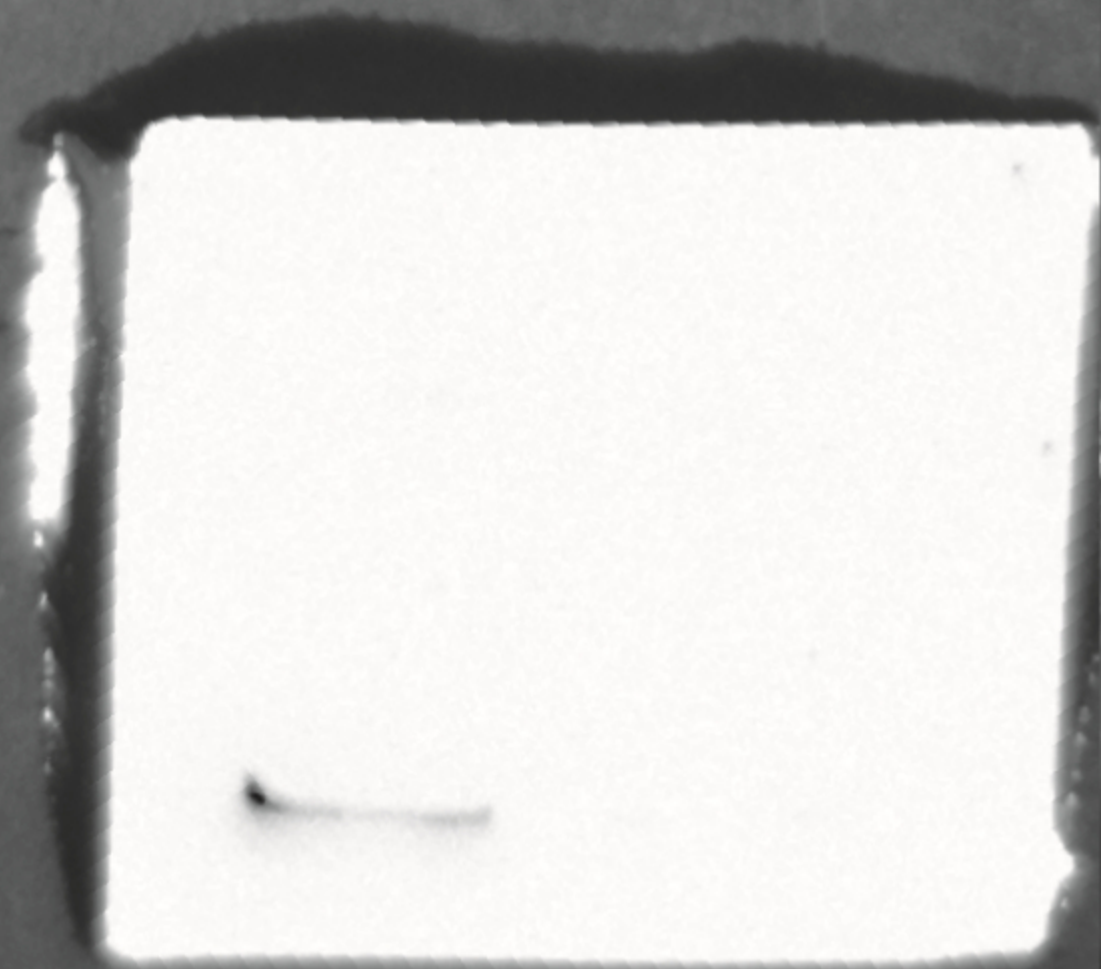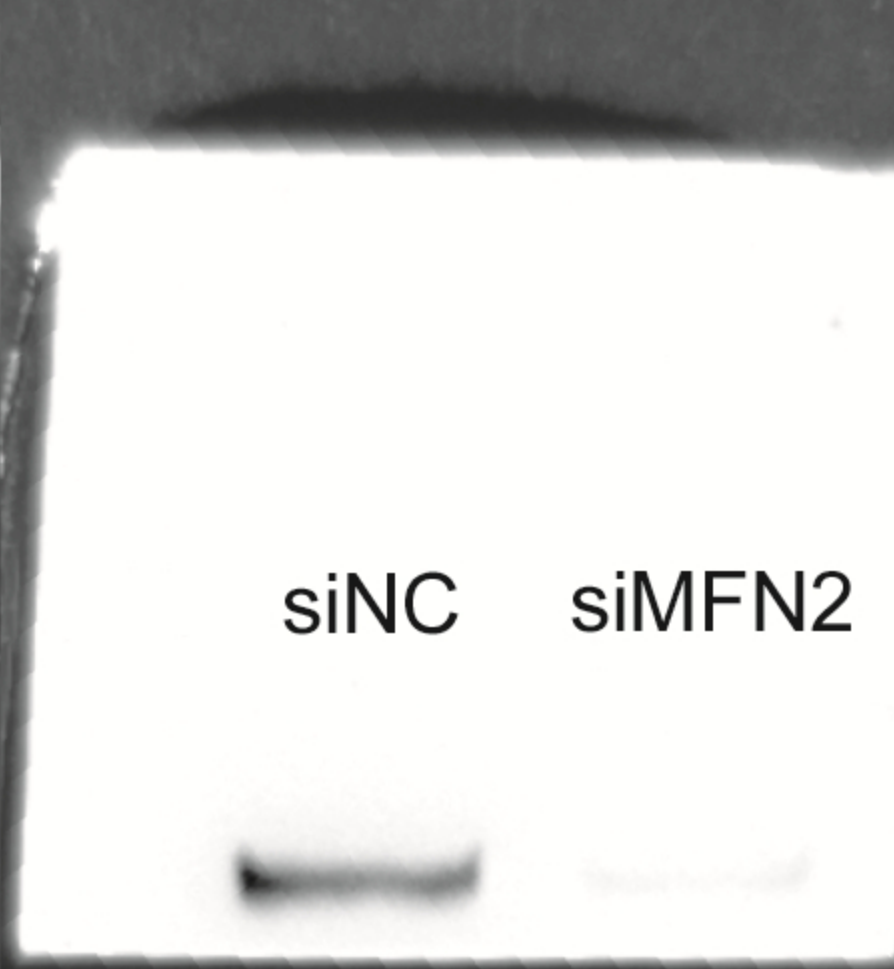

siNC

siMFN2

IB: MFN2

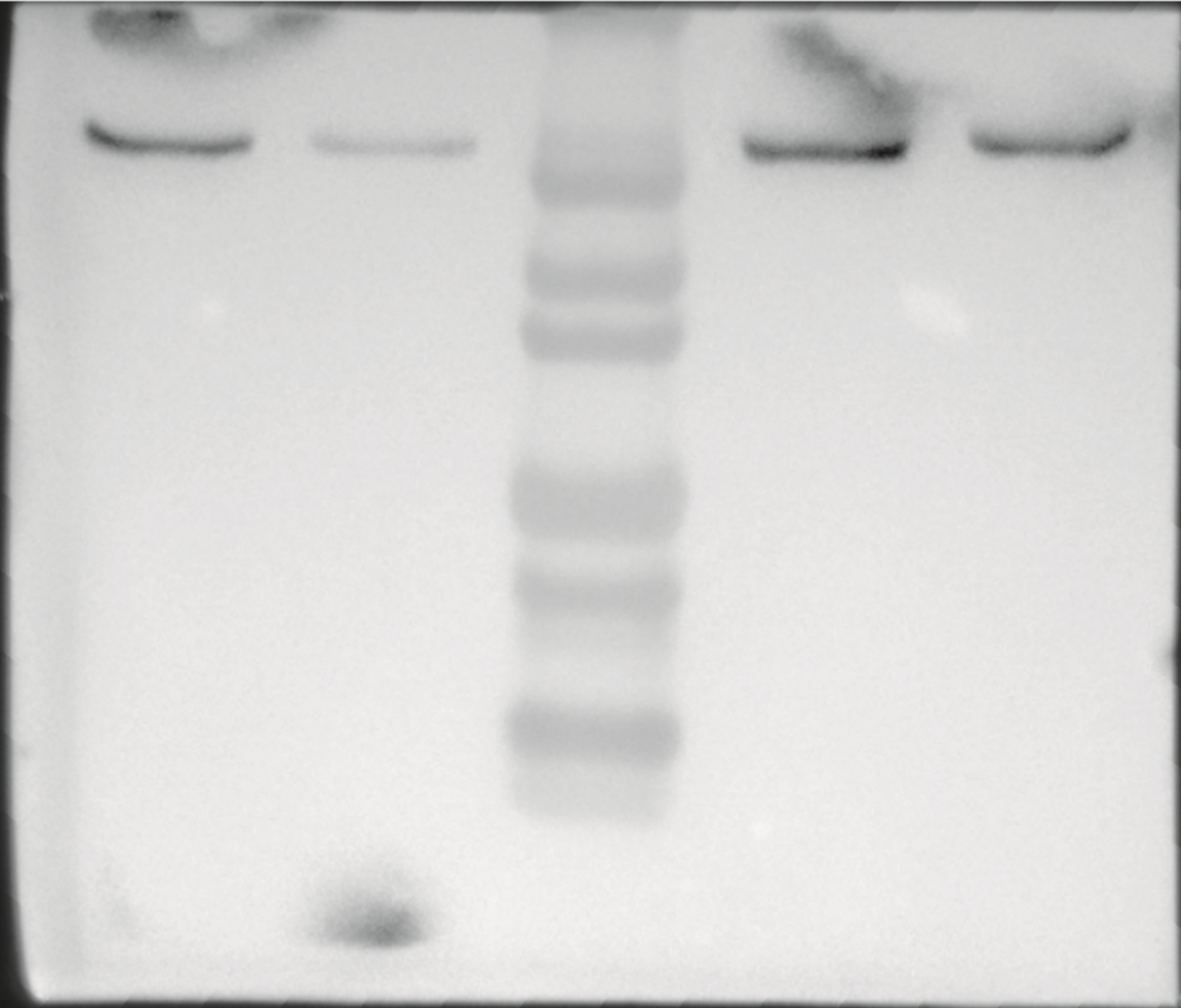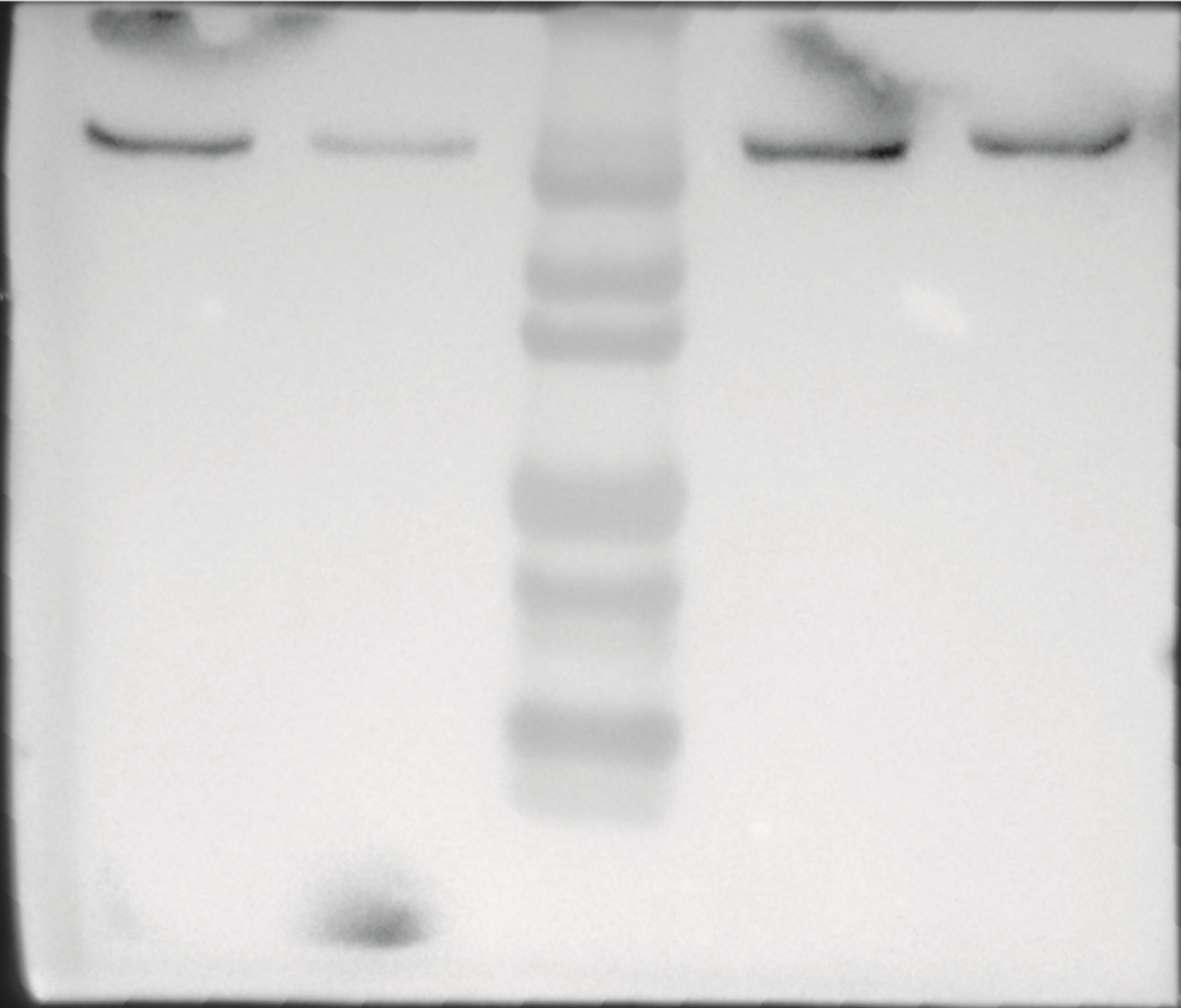

IB: tubulin

Supplement: Supplementary file 12 — Full length WB of figureS5- (A+B) -2 [file 41420_2025_2575_MOESM12_ESM.pdf]

siNC

siMFN1

IB: MFN1

IB: TUBULIN

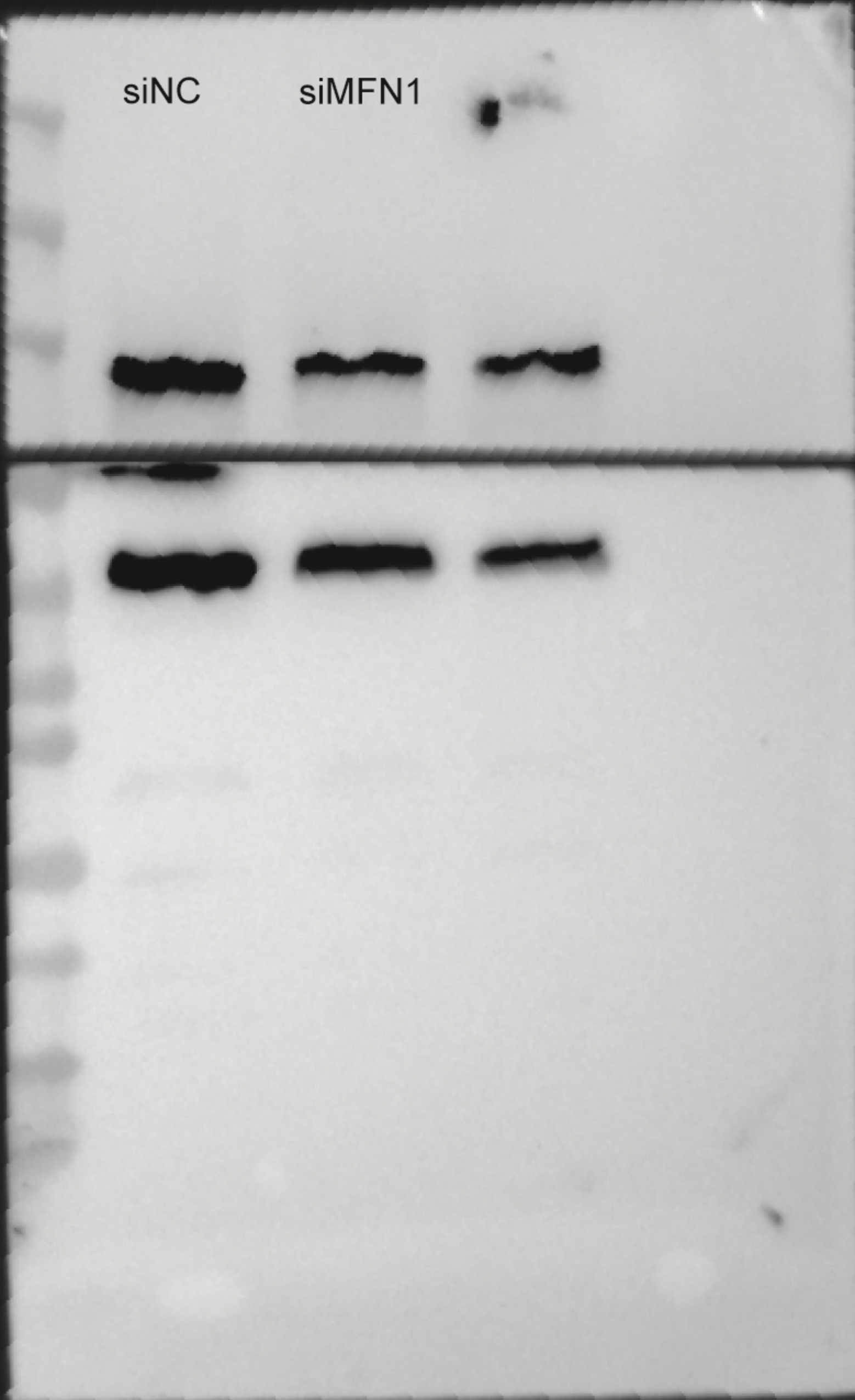

Supplement: Supplementary file 13 — Full length WB of figureS5- (A+B) -3 [file 41420_2025_2575_MOESM13_ESM.pdf]

MFN1

siNC

siMFN1

100KD

siMFN2

siNC

MFN2

70KD

GAPDH

50KD

40KD

35KD

25KD

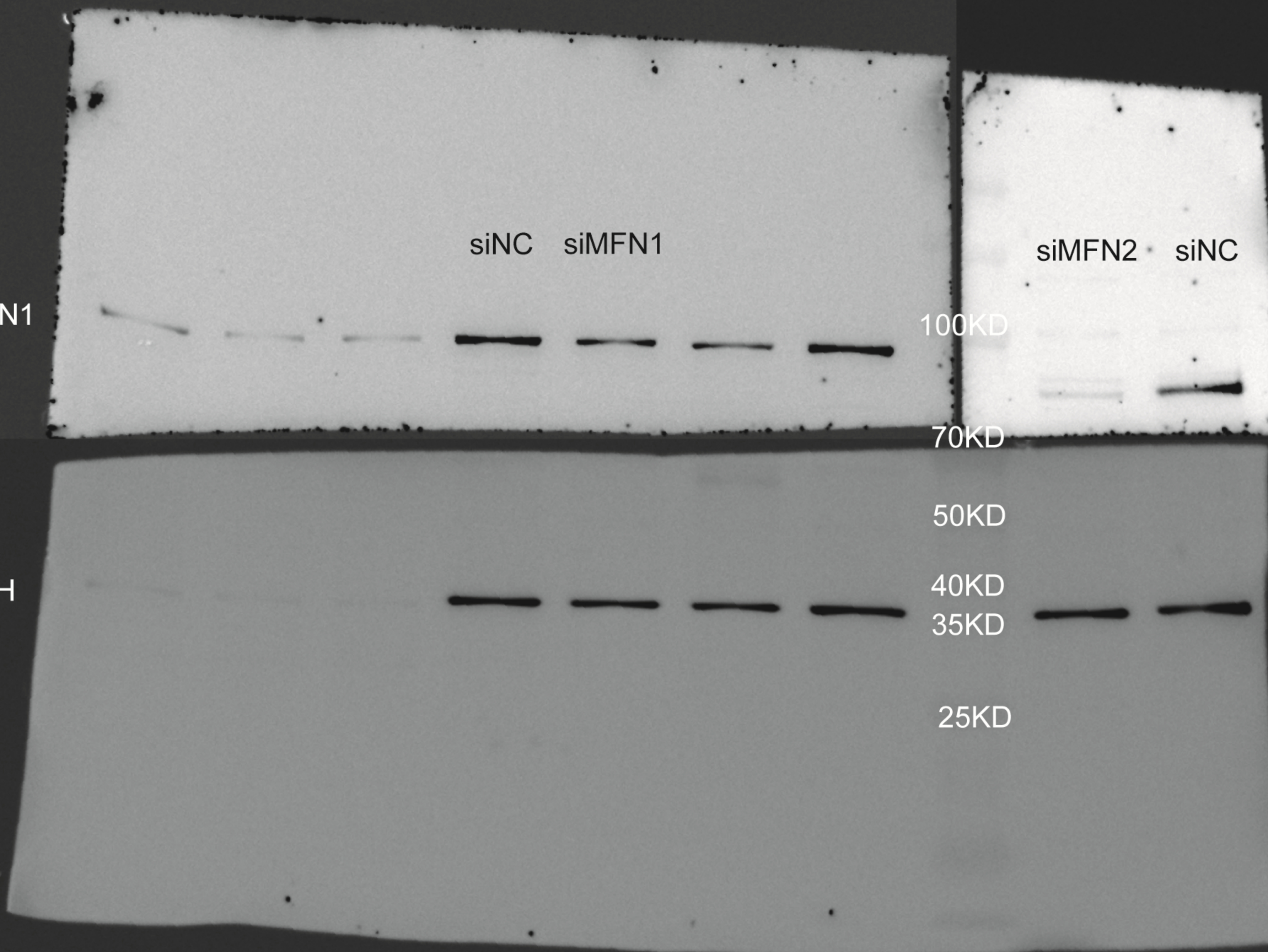

Supplement: Supplementary file 14 — Full length WB of figureS5-C+D [file 41420_2025_2575_MOESM14_ESM.pdf]

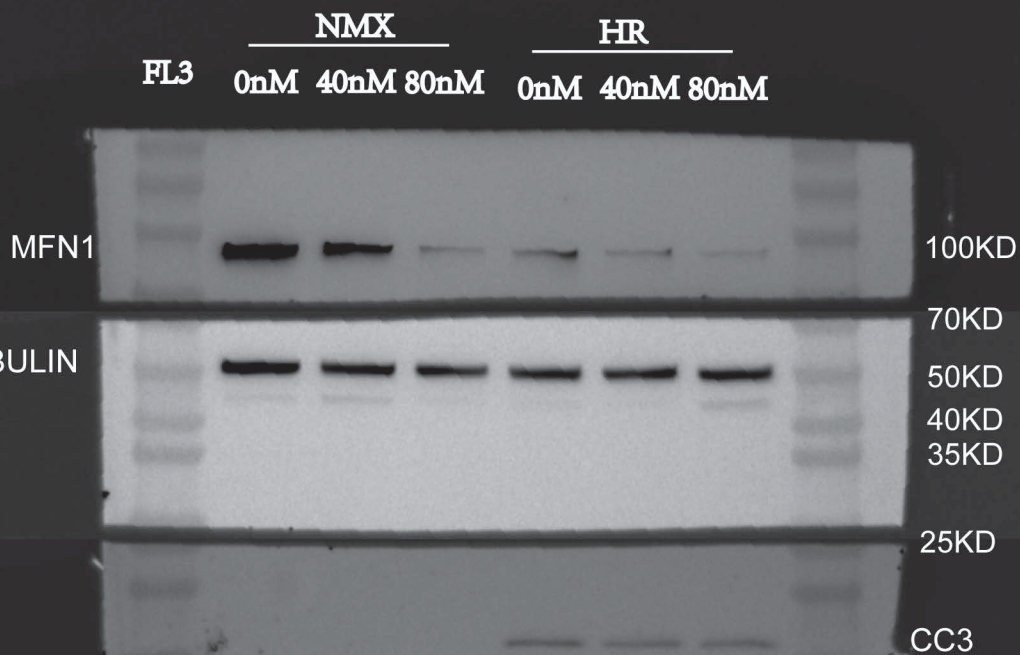

Supplement: Supplementary file 15 — Full length WB of figureS5-E [file 41420_2025_2575_MOESM15_ESM.pdf]
