# Supplementary material for: FL3 mitigates cardiac ischemia-reperfusion injury by promoting mitochondrial fusion to restore calcium homeostasis
Source: Cell Death Discov. 2025 Jul 3;11:304. doi: 10.1038/s41420-025-02575-w (PMC12229567; doi:10.1038/s41420-025-02575-w)
Supplement: Supplementary file 16 — methods [file 41420_2025_2575_MOESM16_ESM.docx]

**MATERIALS AND METHODS**

**Establishment of the Ischemia-Reperfusion (IR) Injury Model and Drug Administration**

Mice in the drug pretreatment group received an intravenous injection of a phosphate buffered saline (PBS) solution containing 0.8 mg/kg Flavagline3 (FL3) via the tail vein for 2 hours before the surgery. Similarly, control group mice received a PBS solution containing Dimethyl sulfoxide (DMSO) at the same concentration. In the post-treatment group, mice were injected with the PBS solution containing 0.8 mg/kg FL3 after releasing the ligature. IR injury modeling was performed on both treatment approaches. Adult mice (8 weeks old) were anesthetized with isoflurane via tracheal intubation, followed by thoracotomy and ligation of the left anterior descending (LAD) artery. After observing the heart turning pale, the chest was closed. One hour later, a second thoracotomy was performed, and the ligature was released. Successful modeling was indicated by the heart turning from pale to red.

**Evan’s Blue + 2,3,5-Triphenyl Tetrazolium Chloride (TTC) Double Staining**

After 24 hours of reperfusion, thoracotomy was performed again on the mice, and the LAD was ligated at the same level as the previous day. Immediately afterward, the area outside the area at risk (AAR) was stained by retrograde perfusion of 0.28% Evan's blue dye through the aorta. The heart was then excised, frozen at -80 °C for 10 minutes, and sliced. The slices were then stained with 1% TTC dye to measure the unstained infarct region. After incubating at 37 °C for 30-60 minutes, the stained myocardial tissue was observed under a microscope. Infarct size (IS) and left ventricular areas were determined by planimetry using Image J. Infarct size was calculated as infarct size divided by the area at risk (IS/AAR).

**Serum Lactate Dehydrogenase (LDH) Measurement**

Mice were anesthetized with pentobarbital (100 mg/kg, intraperitoneally) and heparinized. Blood samples were collected by cutting the carotid artery, centrifuged for 10 minutes at 3,000 rpm to obtain serum, and LDH levels were measured using an LDH assay kit.

**Terminal deoxynucleotidyl Transferase (TdT) dUTP Nick-End Labeling (TUNEL) assay**

For the TUNEL assay, tissue samples were collected after IR modeling, which was performed as previously described. Freshly excised tissue was fixed in 4% buffered formalin at 4 °C for 24 hours. The fixed tissue was automatically embedded in paraffin. Sections were heated at 60 °C and deparaffinized with xylene (2x, 30 minutes each), followed by a series of descending ethanol concentrations (100% ethanol, 1x 30 minutes; 95% ethanol, 1x 30 minutes; 85% ethanol, 1x 30 minutes; 75% ethanol, 1x 30 minutes), and finally rinsed three times in PBS. Heat-induced epitope retrieval was performed on tissue sections in epitope retrieval buffer in a microwave oven for 15 minutes at 100 °C. Subsequently, sections were blocked with 3% bovine serum albumin (BSA) + 20% fetal bovine serum (FBS) at room temperature for 60 minutes. The tissue sections were then incubated overnight at 4 °C with anti-γH2AX rabbit monoclonal antibody. After washing with PBS (3x, 2 minutes each), the sections were incubated with a secondary antibody and washed again with PBS (3x, 2 minutes each). For counterstaining, 4'-6-diamidino-2-phenylindole (DAPI) counterstain reagent was used to stain nuclei. Finally, the slides were mounted with a coverslip mounting medium.

**Echocardiography**

Echocardiographic analysis was performed at the experimental endpoint using a Vevo2100 digital imaging system under 1.5% isoflurane anesthesia. Mid-ventricular M-mode measurements were acquired in the parasternal short-axis view at the level of the papillary muscles. After the mice were acclimated to the anesthesia, images were stored in digital format for review and analysis. Measurements of the left ventricular internal end-diastolic diameter (LVIDd) were made at the time of apparent maximal left ventricular diastolic dimension, and measurements of the left ventricular internal end-systolic diameter (LVIDs) were taken at the time of the most anterior systolic excursion of the posterior wall. Left ventricular ejection fraction (LVEF) was calculated by the cubic method: LVEF (%) = ((LVIDd)³ - (LVIDs)³) / (LVIDd)³ × 100, and left ventricular fractional shortening (FS) was calculated by FS (%) = (LVIDd - LVIDs) / LVIDd × 100. Data were averaged from four cardiac cycles.

**Hematoxylin and Eosin (HE) Staining and Masson's Trichrome Staining**

After 4 weeks of reperfusion, hearts were harvested. The heart tissues were fixed in 4% paraformaldehyde in 0.1 M phosphate buffer for 48 hours, dehydrated, and embedded in paraffin. Sections were cut at 4-µm thickness and mounted on glass slides. HE staining and Masson's trichrome staining were used to assess the extent of fibrosis in cardiac muscle.

**Primary Cardiomyocytes Isolation**

Primary neonatal cardiomyocytes were prepared from 1-day-old Sprague Dawley rat pups using a combination of manual dissection and enzymatic digestion methods. The hearts were excised and immediately placed in PBS. After removing blood and connective tissue, the ventricles were minced. Overnight digestion at 4 °C was performed using pancreatin. For enzymatic digestion, the tissue was subjected to hand shaking at 37 °C with collagenase II (Yeasen, Shanghai, China), repeating the process five times for 8 minutes each. The resulting cell suspension was then filtered through a 70 μm cell strainer and centrifuged to collect the cell pellet. The cells were resuspended in Dulbecco's modified Eagle's medium (DMEM) supplemented with 10% FBS and 1% penicillin-streptomycin (P/S). Subsequently, the cells were cultured at 37 °C in a 5% CO_2_ incubator for 2 hours to allow for differential adhesion and subsequent separation of cardiomyocytes and fibroblasts. After removing the supernatant and centrifuging the cells, they were resuspended in DMEM containing 10% FBS, 1% P/S, and 0.1 mM bromodeoxyuridine (BrdU). These cells were then seeded onto gelatin-coated dishes or coverslips. Neonatal rat ventricular myocytes (NRVMs) were cultured overnight in an incubator at 37 °C and 5% CO_2_. Following 24 hours, the culture medium was replaced with high-glucose medium containing 10% FBS, 1% P/S, and 0.1 mM BrdU. Once the primary cardiomyocytes adhered to the culture surface, they were ready for subsequent experiments.

**Hypoxia/Reoxygenation of Cardiomyocytes**

To induce hypoxia in cardiomyocytes, NRVMs were cultured in DMEM/F12 containing 10% FBS for 48 hours. The medium was then replaced with serum-free, glucose-free medium saturated with 95% N2/5% CO_2_, and the cells were placed in a 37 °C airtight chamber saturated with 95% N2/5% CO_2_ for 24 hours. For normoxic controls, the medium was changed to DMEM/F12, and the cells were incubated at 37 °C /5% CO_2_. When reoxygenation was performed, the medium was replaced with DMEM/F12, and the cells were returned to the normoxic incubator for an additional 2 hours. For the HL-1 cardiomyocyte cell line, the protocol involved 12 hours of hypoxia followed by 2 hours of reoxygenation.

**Western Blot**

Cells were rinsed with ice-cold PBS three times and lysed for approximately 30 minutes. The pyrolysis solution was composed of mammalian cell lysis buffer (MCLB; 50 mM Tris, pH 7.5; 150 mM NaCl; 0.5% NP40), complete ethylenediamine tetraacetic acid (EDTA)-free protease inhibitor (Roche), 1 mM phenylmethylsulfonyl fluoride (PMSF, Amresco), and phosphatase inhibitor cocktail (Roche, USA). The cell lysate was centrifuged at 13,000 × g for 20 minutes, and then protein concentrations were measured by Quick Start™ Bradford (Bio-Rad, USA). Total protein (25 µg) was separated by 10% sodium dodecyl sulfate (SDS)–polyacrylamide gels using an electrophoresis apparatus (BioTanon, Shanghai, China) and transferred to nitrocellulose filter membrane (Millipore, USA). The membranes were blocked with 5% skim milk for 2 h and incubated with the indicated primary antibodies overnight at 4 °C. Next, the membranes were washed three times with Tris-buffered saline with Tween-20 (20 mM Tris, pH 7.4; 137 mM NaCl; 0.05% Tween-20) and then incubated with secondary antibodies at room temperature for 40 minutes. Ultimately, the immunoreactive protein bands were detected by enhanced chemiluminescence using the ChampChemi imaging system (Sage Creation Science).

**Annexin V/Propidium iodide (PI) Double Staining Assay**

Cell apoptosis analysis was performed using PI and fluorescein isothiocyanate (FITC)-labeled Annexin V double staining method. After digestion of the cells with EDTA-free trypsin, cells were collected and washed with precooled PBS. The cells were then resuspended in 100 µL of 1× Binding Buffer, followed by the addition of 5 µL of Annexin V-FITC and 10 µL of PI Staining Solution, and gently mixed. The reaction was allowed to proceed in the dark at room temperature for 10-15 minutes. Flow cytometry analysis was conducted to differentiate apoptotic cells (Annexin V-positive and PI-negative) from necrotic cells (Annexin V-positive and PI-positive). Data were analyzed using FlowJo software.

**5,5′,6,6′-Tetrachloro-1,1′,3,3′-Tetraethylbenzimidazolocarbo-cyanine Iodide (JC-1) Staining**

HL-1 cells were stained with JC-1 (cat. no. T3168; ThermoFisher) at 37 °C for 30 minutes, washed three times with PBS, and analyzed by flow cytometry (BD FACSCanto™; BD Biosciences). Data were processed using FlowJo version ×0.7 (FlowJo LLC). In healthy mitochondria, JC-1 forms aggregates in the matrix, emitting red fluorescence (Excitation 585 nm; Emission 590 nm). In damaged mitochondria, where membrane potential declines or is lost, JC-1 remains as monomers in the cytoplasm, emitting green fluorescence (Excitation 514 nm; Emission 529 nm). The ratio of phycoerythrin (PE)-H (red) to fluorescein isothiocyanate (FITC)-H (green) indicates changes in mitochondrial membrane potential.

**RNA Interference**

Small-interfering RNA (siRNA) oligos targeting mitofusin1/2 (MFN1/2) were synthesized and annealed by General Biol (Shanghai, China). These fragments were designed to target MFN1 and MFN2 transcripts. The siMFN1 sequences were as follows: rat-derived siMFN1 sense: 5’-AUACAGGGCUACAGAAACATT-3’; rat-derived siMFN2 sense: 5’-CCUCAAGGUUUAUAAGAAUTT-3’; human-derived siMFN1 sense: 5’-GCUGGAUAGCUGGAUUGAUAAGUTT-3’; human-derived siMFN2 sense: 5’-GGAAGAGCACCGUGAUCAATT-3’; mouse-derived siMFN1 sense: 5’-AUACAGGGCUACAGAAACATT-3’; siNC sense: 5’-UUCUCCGAACGUGUCACGUTT-3’. Cells were transfected using RNAiPro Transfection Reagent (MIKX) according to the previously described protocol.

**Transmission Electron Microscopy**

Transmission electron microscopy (TEM) experiments were conducted at the Electron Microscopy Platform of the Core Facility of Basic Medical Sciences, Shanghai Jiao Tong University School of Medicine, and the Chinese Academy of Sciences Shanghai Branch. Myocardial tissue from IR-injured mice was collected and sectioned into slices less than 1 mm thick. Fresh tissue sections were immersed in a TEM fixation solution for observation, fixed at 4°C for 4 hours, and subsequently treated with 1% osmium tetroxide for 120 minutes. The sections were then dehydrated using graded alcohol, embedded, and cut into ultrathin sections. Finally, the ultrastructure of MAMs was imaged using TEM.

**GCaMP6 Calcium Ion Assay**

To quantify calcium ions in different subcellular compartments, GCaMP6 was expressed in HeLa cells via lentiviral infection to create cell lines localized to the mitochondrial matrix, intermembrane space (IMS), mitochondria-associated endoplasmic reticulum membranes (MAM), endoplasmic reticulum (ER), and cytoplasm. Additionally, NRVMs were infected to establish cells with GCaMP6 localized to the mitochondrial matrix expression. dTomato was co-expressed in the nucleus to normalize the GCaMP6 signal. Signals from GCaMP6 and dTomato were measured in the FITC and PE channels of the flow cytometer (Beckman, CytoFlex S). For calcium flux measurements, live-cell imaging of GCaMP6-expressing cells was conducted at 37 °C and 5% CO₂ using a fluorescence microscope (LEICA, DMi8). After recording baseline fluorescence, histamine was added to induce calcium release from the ER. Time-lapse images were collected every 2 seconds for 10 minutes, and fluorescence intensity was analyzed using Fiji-ImageJ.

**RNA Isolation and Quantitative Real-Time PCR**

Total RNA was extracted using a cellular RNA extraction reagent (Suzhou Yingze Biotechnology Co., Ltd., China) following the manufacturer’s instructions. Subsequent experiments were performed with qPCR SYBR Green Master Mix (7E2312J4, Vazyme, USA) on a LightCycler® 96 Instrument real-time PCR system (Roche, USA). All data were normalized to α-tubulin expression and expressed as fold change. Primer sequences were as follows: MFN1 (Forward: 5’-ATCACTGCAATCTTCGGCCA-3’, Reverse: 5’-AGCAGTTGGTTGTGTGACCA-3’), MFN2 (Forward: 5’-CGAGGCTCTGGATTCACTTCA-3’, Reverse: 5’-CCAACCAGCCAGCTTTATTCC-3’), OPA1 (Forward: 5’-CCTTTGTCGCAGAGGTTTTTATTAC-3’, Reverse: 5’-CATTGCATTCAGCTCAGAATC-3’), and α-tubulin (Forward: 5’-ATGCGCGAGTGCATTTCAG-3’, Reverse: 5’-CACCAATGGTCTTATCGCTGG-3’).

**Nuclear-Cytoplasmic Fractionation and Western Blot Analysis**

Nuclear and cytoplasmic proteins were extracted using a commercial protein extraction kit (Beyotime, China) following the manufacturer’s protocol. Briefly, nuclear cytoplasmic extraction buffer supplemented with protease inhibitor cocktail was used to isolate nuclear fractions. For Western blotting, 25 μg of cytosolic protein, or 25 μg of nuclear protein were separated by SDS-PAGE and transferred to NC membranes. Target proteins were detected using specific primary antibodies, followed by HRP-conjugated secondary antibodies.

**Statistical Analysis**

All data are presented as mean ± standard error of the mean (SEM). Statistical significance was determined using [insert specific tests, e.g., Student's t-test for two-group comparisons, ANOVA for multiple comparisons] as appropriate. A p value of <0.05 was considered statistically significant. Analyses were conducted using GraphPad Prism.
